# Anti‑Inflammatory Sesquiterpenoids From Two Asteraceae Plants Native to Kazakhstan: Isolation, Structural Elucidation, and Mechanistic Insights

**DOI:** 10.1002/cbdv.71683

**Published:** 2026-09-20

**Authors:** Arailym Zhomartova, Wen‐Jing Gao, Janar Jenis, Chu‐Hong Fang, Pei‐Qian Wu, Jian‐Min Yue, Bin Zhou

**Affiliations:** ^1^ Ethnomedicine and Biofunctional Molecule Research Center State Key Laboratory of Drug Research Shanghai Institute of Materia Medica Chinese Academy of Sciences Shanghai China; ^2^ University of Chinese Academy of Sciences Beijing China; ^3^ The Research Center for Medicinal Plants Al‐Farabi Kazakh National University Almaty Kazakhstan; ^4^ Shandong Laboratory of Yantai Drug Discovery Bohai Rim Advanced Research Institute for Drug Discovery Yantai Shandong Republic of China

**Keywords:** anti‐inflammation, *Artemisia gracilescens*, asteraceae, mechanistic studies, sesquiterpenoids, *Taraxacum tianschanicum*

## Abstract

Traditionally used in Kazakh folk medicine, the Asteraceae species *Taraxacum tianschanicum* and *Artemisia gracilescens* were investigated for their sesquiterpenoid profiles despite being rich in flavonoids and fatty acids, leading to the isolation of 10 sesquiterpenoids of three structural types. These include one previously undescribed germacrane (**1**) and two known germacranes (**2** and **3**) from *T. tianschanicum*, as well as two germacranes (**4** and **5**), one guaiane (**6**), and four eudesmanes (**7–10**) from *A. gracilescens*. The structures of these compounds were established through comprehensive analysis of the spectroscopic data and electronic circular dichroism (ECD) calculations. Several compounds exhibited significant anti‐inflammatory effects (IC_50_ = 1.15–8.37 µM) with low cytotoxicity, and mechanistic studies on structurally distinct sesquiterpenoids **4** (germacrane‐type) and **8** (eudesmane‐type) revealed that both suppress IL‐6, IL‐1β, iNOS, and COX‐2. Notably, unlike compound **4**, the activity of **8** involved suppression of the MAPK pathway, indicating a clear mechanistic divergence.

## Graphical abstract

This study identifies potent anti‐inflammatory sesquiterpenoids from two Asteraceae plants native to Kazakhstan. Among 10 isolated compounds, both **4** and **8** inhibit key inflammatory markers, but only **8** acts by suppressing the MAPK pathway, indicating a structure‐dependent mechanistic divergence.

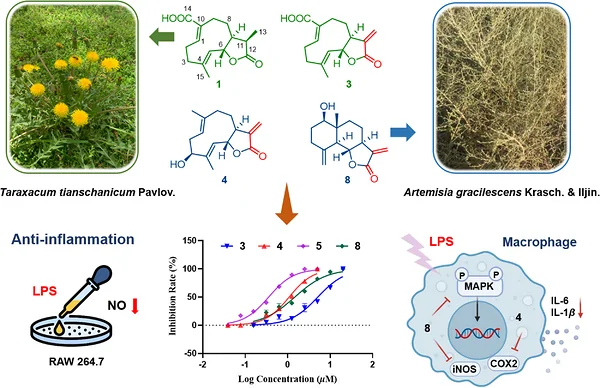

## 1 Introduction

Kazakhstan's flora is characterized by a significant diversity of medicinal plants, encompassing approximately 1406 species from 134 families [1]. Among these, the Asteraceae family stands out as the most species rich, with about 196 species that are an important source of biologically active compounds, especially sesquiterpenoids with a broad spectrum of pharmacological activity [2, 3]. Within this family, *Taraxacum* and *Artemisia* are of particular importance. *Taraxacum* consists of species commonly known as dandelions, which have long been used for nutritional and therapeutic purposes [4, 5]. Species of *Artemisia* play a vital role in both traditional and modern medicine, most notably *Artemisia annua*, the source of the well‐known antimalarial sesquiterpene lactone artemisinin [6, 7]. In the present study, two plant species, *T. tianschanicum* and *A. gracilescens*, were selected for investigation owing to their documented applications in Kazakh folk medicine and convenient availability. Up to now, no phytochemical study has been conducted on *T. tianschanicum*, while only a few sesquiterpenoids have been reported from *A. gracilescens* [3]. Thereby, the chemical composition and pharmacological profile of these two species remain largely unexplored, warranting further investigation.

**FIGURE 1:**
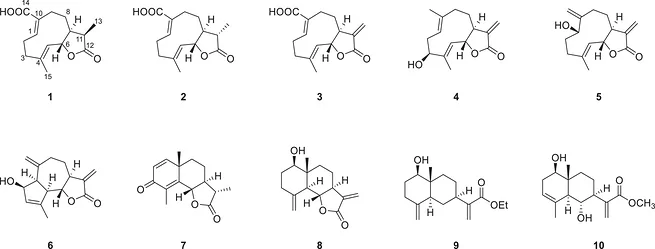
Structures of compounds **1–10**.

Plants from the mountainous regions of southeastern Kazakhstan are subjected to considerable cold stress, with temperatures frequently dropping below 0°C. This stress induces a significant reprogramming of metabolic networks as an adaptive strategy, frequently leading to the marked accumulation of flavonoids and fatty acids, a finding corroborated by our initial metabolomic screening (Table S1) [8, 9, 10]. However, given the taxonomic significance of sesquiterpenoids in the plant species under investigation, this study specifically focused on the isolation and characterization of these constituents. Consequently, 10 sesquiterpenoids belonging to three structural classes, including five germacranes (**1**–**5**), one guaiane (**6**), and four eudesmanes (**7**–**10**), were isolated from the whole plants of *T. tianschanicum* and *A. gracilescens* (Figure 1).

The structure of previously undescribed compound **1** was elucidated through comprehensive analysis of its spectroscopic data and electronic circular dichroism (ECD) calculations. Furthermore, cytotoxic and anti‐inflammatory assays revealed some compounds demonstrated significant anti‐inflammatory potency, with IC_50_ values ranging from 1.15 to 8.37 µM, while showing low cytotoxicity. Herein, we present a full account of the isolation, structural elucidation, and biological evaluation of these compounds.

## 2 Results and Discussion

**FIGURE 2:**
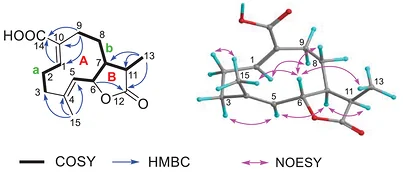
Key 2D NMR correlation of compound **1**.

Compound **1** gave a molecular formula of C_15_H_20_O_4_, as established by (−)‐HRESIMS peak at *m*/*z* 263.1288 [M ‒ H]^‒^ (Figures S7 and S8) and its ^13^C NMR data, requiring six degrees of unsaturation. The IR spectrum (Figure S9) showed absorption bands for the carbonyl (1767, 1704 cm−^1^) groups. Interpretation of the ^1^H and ^13^C NMR (Table 1; Figures S1 and S2) showed six sp^2^‐hybridized carbon signals, attributed to two carbonyls (*δ*_C_ 180.0 and 172.9) and two trisubstituted double bonds (*δ*_H_ 5.78, 4.86; *δ*_C_ 150.2, 141.5, 130.1, 126.4). The remaining carbon resonances were resolved into four methylenes, three methines (one oxygenated *δ*_H_ 4. 78; *δ*_C_ 81.1), and two methyls, with the aid of DEPT and HSQC data (Figure S3). These findings suggested compound **1** was a dicyclic sesquiterpenoid, structurally related to 11*β*, 13‐dihydrotaraxinic acid (**2**) [11, 12]. A detailed comparison of the ^1^H and ^13^C NMR data (Table 1) suggested compounds **1** and **2** shared the same planar structure. Specifically, analysis of the ^1^H–^1^H COSY correlations (Figures 2 and S5) delineated two spin–spin coupling fragments a and b, as illustrated in bold bonds. Key HMBC cross‐peaks (Figures 2 and S4) from H_3_‐15 to C‐3, C‐4 (*δ*_C_ 141.5), and C‐5 (*δ*_C_ 126.4), H‐1 (*δ*_H_ 5.78) to C‐14 (*δ*_C_ 172.9), H_2_‐9 to and C‐1 (*δ*_C_ 150.2), C‐10 (*δ*_C_ 130.1), and C‐14 connected these fragments into a ten‐membered A ring, bearing double bonds at C‐4/C‐5 and C‐1/C‐10, and a carboxyl group at C‐10.

**TABLE 1:** ^1^H (600 MHz) and ^13^C NMR (125 MHz) data of compounds **1** and **2**.

Position	1^a^		2		
	*δ*_C_, type	*δ*_H_, mult. (J in Hz)	*δ*_C_ ^b^ type	*δ*_H_ ^b^ mult. (J in Hz)	*δ*_H_ ^a^ mult. (J in Hz)
1	150.2, CH	5.78, dd (12.5, 4.3)	146.9, CH	5.68, dd (12.2, 3.5)	5.76, dd (12.9, 4.3)
2*α*	26.6, CH_2_	2.34, overlapped	27.5, CH_2_	2.28, overlapped	2.35, overlapped
2*β*	—	3.40, m	—	3.30, overlapped	3.38, m
3*α*	39.4, CH_2_	2.26, m	40.1, CH_2_	2.28, overlapped	2.26, overlapped
3*β*	—	2.36, overlapped	—	2.35, overlapped	2.35, overlapped
4	141.5, C	—	142.9, C	—	—
5	126.4, CH	4.86, d (10.1)	127.4, CH	4.90, dd (10.1, 1.5)	4.84, d (10.1)
6	81.1, CH	4.78, t (9.8)	83.4, CH	4.72, dd (10.1, 9.2)	4.59, t (9.7)
7	49.8, CH	2.17, m	55.8, CH	1.94, overlapped	1.93, overlapped
8*α*	27.3, CH_2_	1.91, overlapped	31.4, CH_2_	1.94, overlapped	1.93, overlapped
8*β*	—	1.78, m	—	1.77, overlapped	1.66, m
9*α*	37.0, CH_2_	1.92, overlapped	37.8, CH_2_	1.94, overlapped	1.93, overlapped
9*β*	—	2.88, m	—	2.80, m	2.88, m
10	130.1, C	—+	133.0, C	—	—
11	41.2, CH	2.67, dq (7.7, 7.7)	43.5, CH	2.36, dq (12.3, 7.0)	2.24, dq (12.3, 7.0)
12	180.0, C	—	181.4, C	—	—
13	11.0, CH_3_	1.21, d (7.7)	13.2, CH_3_	1.21, d (7.0)	1.26, d (7.0)
14	172.9, C	—	171.6, C	—	—
15	16.9, CH_3_	1.62, br s	17.0, CH_3_	1.64, d (1.5)	1.63, br s

^a^ Measured in CDCl_3._

^b^ Measured in methanol‐d_4_.

Furthermore, considering the remaining one degree of unsaturation, and HMBC cross‐peaks from H_3_‐13 and H‐6 (*δ*_H_ 4.78) to C‐12 (*δ*_C_ 180.0), allowed construction of a γ‑lactone B‑ring via an oxygen linkage between C‑6 and C‑12. Collectively, the planar structure of compound **1**, a germacrane‐type sesquiterpenoid, was elucidated as shown.

**FIGURE 3:**
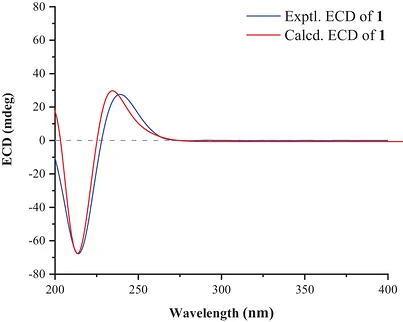
Experimental and calculated ECD spectra of compound **1**.

Comparison of the ^1^H and ^13^C NMR chemical shifts, and coupling constants between compounds **1** and **2** (Table 1) showed distinct differences around C‐11, particularly in the vicinal coupling constant between H‐7 and H‐11 (*J* = 7.7 Hz in **1** vs. *J* = 12.3 Hz in **2**), suggesting they varied in the configuration at C‐11. Further analysis of the NOESY data (Figures 2 and S6) showed cross‐peaks of H‐6/H‐8*β* and H_3_‐13, and H‐7/H‐11 and H‐5, thereby confirming the β‐orientation of H‐6 and H_3_‐13, and the *α*‐configuration of H‐7. The double bonds ∆^4^ and ∆^1(10)^ were assigned *E* and *Z* geometries, respectively, based on the NOESY correlations of H‐5/H‐3*α* and H_3_‐15/H‐6, and H‐1/H‐9*α*. Ultimately, TDDFT‐ECD calculations (Figure 3) at the B3LYP/6‐31G*//ωb97xd/6‐311G** level of theory in methanol with the IEFPCM solvent model assigned the absolute configuration of compound **1** as being 6*S*, 7*S*, 11*R*. Accordingly, the structure of compound **1** was fully elucidated and assigned the name (11*R*)‐11,13‐dihydrotaraxinic acid. Compounds **1** and **2** are a pair of C‐11 epimers, which are speculated to be formed through a regioselective oxidation at C‐12 and C‐13, followed by a cyclization reaction with C‐6.

In addition, nine known compounds, including four germacrane‐type sesquiterpenoids 11*β*,13‐dihydrotaraxinic acid (**2**) [11, 12], taraxinic acid (**3**) [13], hanphyllin (**4**) [14], and artemorin (**5**) [15, 16], one guaiane‐type sesquiterpenoid 2*β*‐hydroxyguaia‐3,10(14),11(13)‐trien‐12,6*α*‐olide (**6**) [17], and four eudesmane‐type sesquiterpenoids *α*‐santonin (**7**) [18], reynosin (**8**) [19], 1*β*,6*α*‐dihydroxycostic acid ethyl ester (**9**) [20], and magnograndin F (**10**) [21] were isolated and identified based on comparison of their 1D NMR and ESIMS data with those reported (Figures 1 and S10–S36). As a note, compounds **1**–**3** were isolated from the species *T. tianschanicum*, while the others were identified from *A. gracilescens*.

**FIGURE 4:**
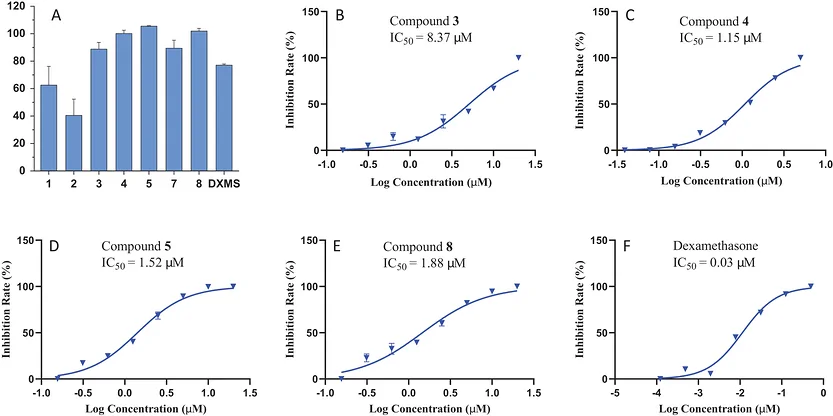
(A) Effects of compounds **1**–**5**, **7**, and **8** on the production of NO in the LPS‐activated RAW 264.7 cells. Data were normalized to the LPS group (0.05 µg/mL) for 24 h. (B–F) IC_50_ values of compounds **3**–**5**, and **8** on LPS‐induced NO production in RAW 264.7 cells. Dexamethasone (DXMS) was used as the positive control. The results were expressed as mean ± standard deviation (SD) from three independent experiments (*n* = 3).

Given the well‐known anti‐inflammatory and cytotoxic potential of sesquiterpenoids, as well as the traditional use of both source species in treating inflammatory ailments, the isolated compounds were evaluated for their anti‐inflammatory and cytotoxic activities [22, 23, 24]. The cytotoxicity assay was also employed to distinguish genuine anti‐inflammatory effects from those arising from general cell toxicity. The cytotoxicity of compounds **1–5**, **7**, and **8** was examined against three cancer cell lines (MDA‐MB‐231, A549, and Hep G2) using the MTT method, and none of them showed obvious cytotoxic effects at 20 µM (Table 2), with growth inhibition remaining below 40%. Then, the anti‐inflammatory activity of these compounds was evaluated in LPS‐induced RAW 264.7 cells, and compounds **3–5**, and **8** showed obvious NO production inhibition at non‐cytotoxic concentrations (Figure 4A; Tables 2 and S2). Dexamethasone (DXMS) serves as a positive control due to its well‐studied mechanism and its role as a reference standard [27]. Subsequent dose–response experiments demonstrated that compound **3**, derived from *T. tianschanicum*, exerted an IC_50_ value of 8.37 ± 0.99 µM (Figure 4B), whereas compounds **4**, **5**, and **8**, sourced from *A. gracilescens* displayed significant activity, with IC_50_ values of 1.15 ± 0.12, 1.52 ± 0.16, and 1.88 ± 0.17 µM, respectively (Figure 4C–E), relative to the positive control DXMS (0.040 ± 0.004 µM). These findings, combined with structural analysis, suggested the presence of an exocyclic double bond  Δ^11(13)^ constituting the α,β‐unsaturated γ‐lactone fragment B is a key structural determinant of this activity.

**TABLE 2:** Inhibitory effects of compounds **1–5**, **7**, and **8** at 20 µM on the proliferation of selected cell lines.

Compd.	MDA‐MB‐231	A549	Hep G2	RAW 264.7
**1**	−2.78	−16.83	−2.06	−2.55
**2**	−5.28	−1.60	−8.55	6.17
**3**	−4.04	−6.56	−2.23	−1.31
**4**	9.94	2.08	36.68	57.66^a^
**5**	−2.29	−0.71	11.14	−8.25
**7**	−7.89	−10.33	−2.67	13.72
**8**	−0.04	−6.64	16.22	−1.78

^a^ Compound **4** exhibited cytotoxicity against RAW 264.7 cells at 20 µM, but no significant toxicity was observed at lower concentrations (1.25–10 µM), with cell viability rates of 106.09%, 99.82%, 87.56%, and 78.72%, respectively.

**FIGURE 5:**
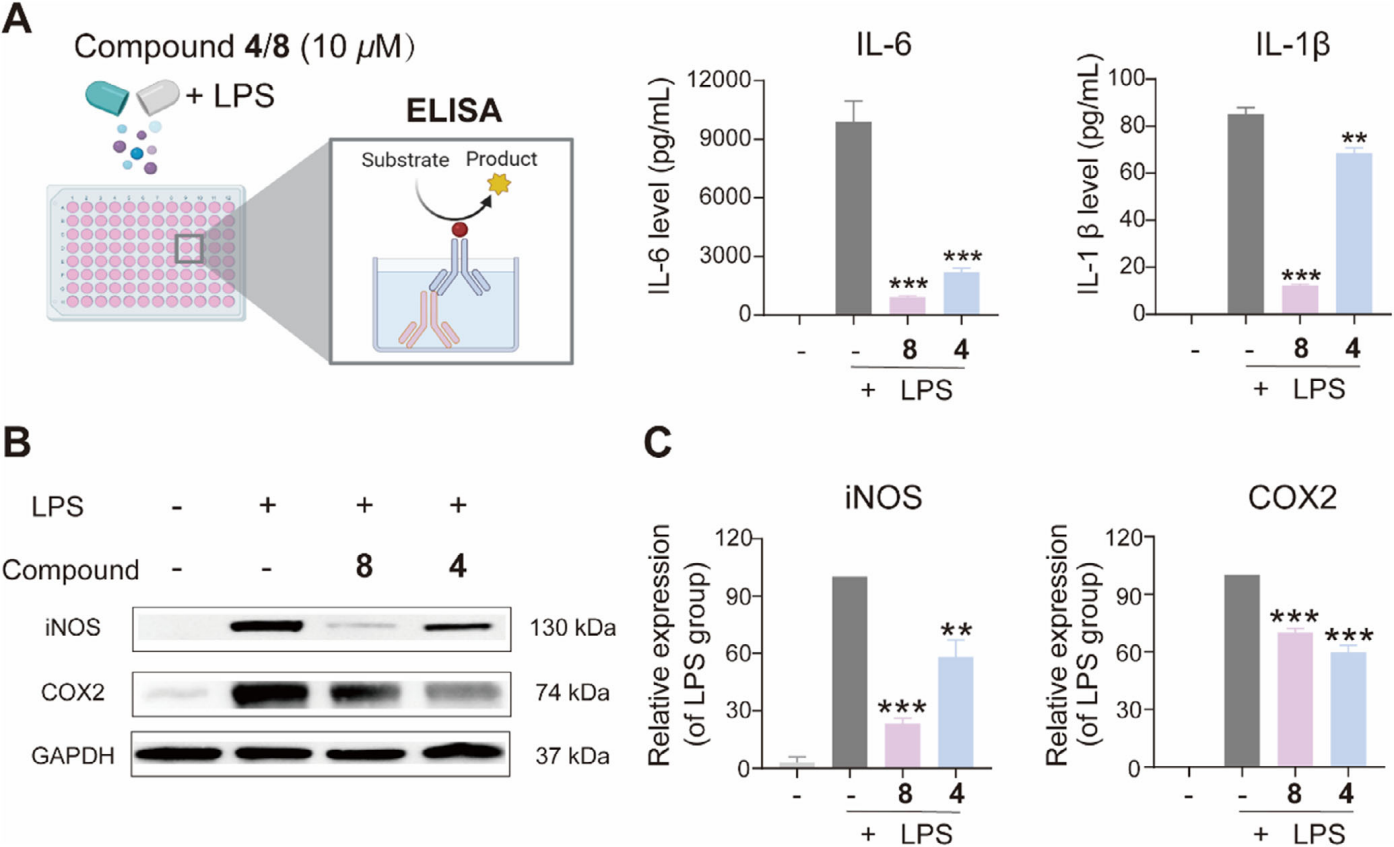
(A) ELISA measurement of IL‐6 and IL‐1β release from LPS‐stimulated RAW 264.7 cells treated with compounds **8** (10 µM) and **4** (10 µM). (B–C) Effects of compounds **8** (10 µM) and **4** (10 µM) on iNOS and COX‐2 expression in LPS‐induced RAW 264.7 cells. (B) Original bands of iNOS and COX‐2. GAPDH was used as an internal control. (C) Relative expression of these proteins. The data are represented as mean ± SD for three independent experiments (*n* = 3), each performed with technical triplicates. Statistical significance was determined using one‐way analysis of variance (ANOVA) followed by Dunnett's post‐hoc test for multiple comparisons against the LPS group (^**^ *p* < 0.01, ^***^ *p* < 0.001 compared with LPS group).

Given their potent anti‐inflammatory activity, compounds **4** and **8**, which are representatives of germacrane and eudesmane sesquiterpenoids, respectively, were selected for a further mechanistic study. The effects of these compounds on the production of the pro‐inflammatory cytokines IL‑6 and IL‑1β were first examined in LPS‐stimulated RAW 264.7 cells using ELISA. As shown in Figure 5A, LPS stimulation dramatically increased the levels of IL‑6 and IL‑1β in the culture supernatant to 9891 and 85.13 pg/mL, respectively. Treatment with compound **8** (10 µM) potently reduced these levels to 914 pg/mL for IL‑6 and 12.31 pg/mL for IL‑1β. Compound **4** (10 µM) also exhibited significant inhibitory activity, lowering IL‑6 and IL‑1β levels to 2180 and 68.52 pg/mL, respectively. In line with the cytokine data, both compounds markedly suppressed the LPS‐induced upregulation of iNOS and COX‑2 protein levels (Figure 5B,C).

**FIGURE 6:**
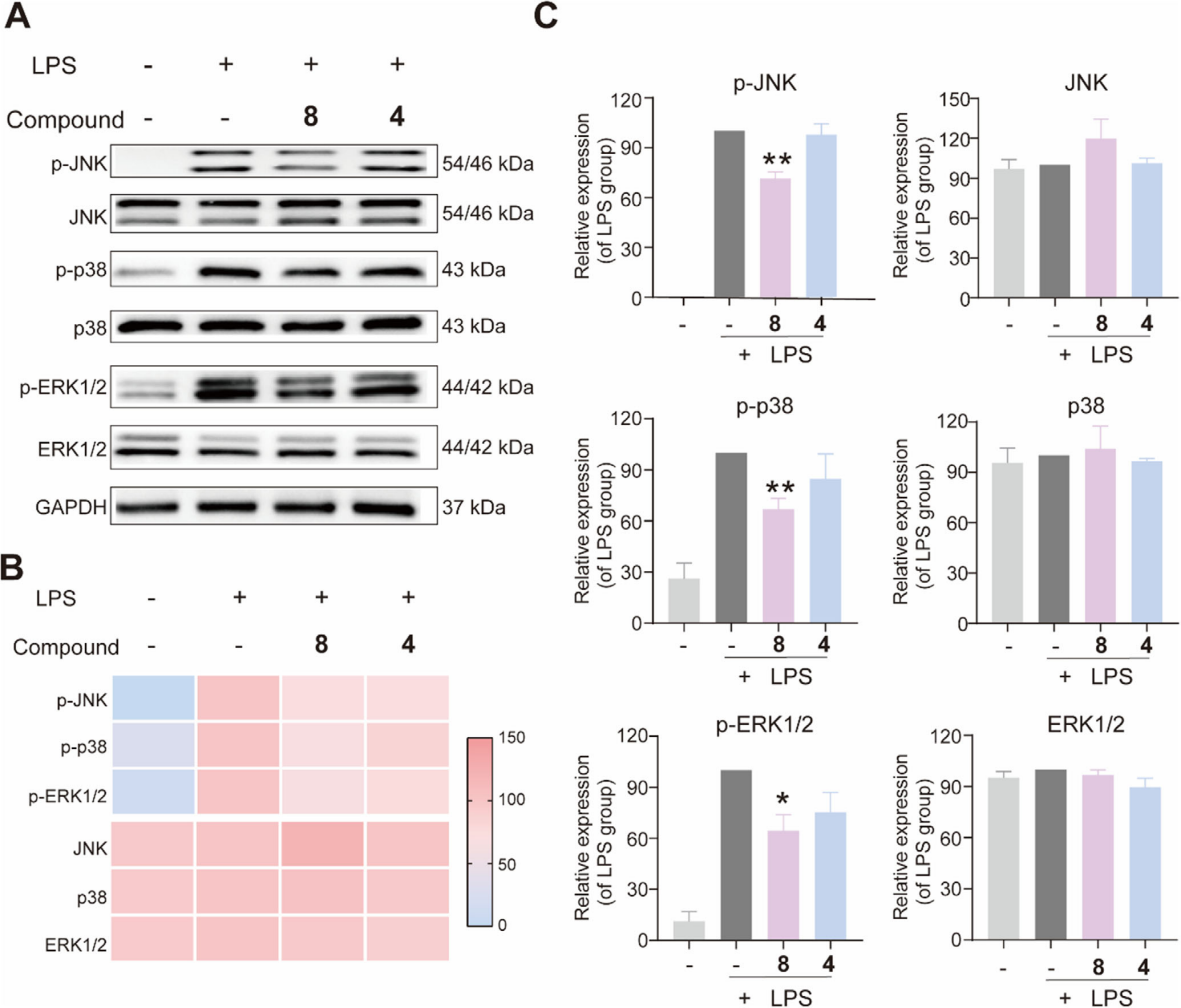
Effects of compounds **8** (10 µM) and **4** (10 µM) on the protein expressions of MAPK signaling pathways in LPS‐induced RAW 264.7 cells. (A) Original bands of phosphor‐JNK, JNK, phosphor‐p38, p38, phosphor‐ERK1/2, and ERK1/2. GAPDH was used as an internal control. (B) Heatmap representation of protein expressions of MAPK signaling pathways under different compounds treatment. (C) Relative expression of these proteins. The data are represented as mean ± SD for three independent experiments (*n* = 3), each performed with technical triplicates. Statistical significance was determined using one‐way analysis of variance (ANOVA) followed by Dunnett's post‐hoc test for multiple comparisons against the LPS group (^*^ *p* < 0.05, ^**^ *p* < 0.01 compared with LPS group).

Considering the pivotal role played by the mitogen‐activated protein kinase (MAPK) signaling pathway in inflammatory responses [25, 26], we further investigated the phosphorylation status of its key components, c‐Jun *N*‐terminal kinase (JNK), p38/MAPK, and extracellular signal‐regulated kinase 1/2 (ERK1/2). As illustrated in Figure 6A–C, LPS stimulation strongly enhanced the phosphorylation of JNK, p38, and ERK1/2 without affecting their total protein levels. Notably, compound **8** significantly inhibited the activation of the MAPK pathway, as reflected by the reduced phosphorylation levels of all three kinases. In contrast, compound **4** did not show a clear inhibitory effect on MAPK phosphorylation under the same experimental conditions. Overall, this differential action suggested that the potent anti‐inflammatory efficacy of compound **8** may be mediated, at least in part, through MAPK pathway inhibition, whereas compound **4** likely exerts its effects via MAPK‐independent mechanisms.

## 3 Conclusions

In conclusion, this study presents the first detailed phytochemical analysis of two species of Kazakh flora, *Taraxacum tianschanicum* and *Artemisia gracilescens*. A total of 10 sesquiterpenoids were isolated, including one new compound (11*R*)‐11,13‐dihydrotaraxinic acid (**1**), while the remaining nine compounds except for compound **7** were obtained from these two plants for the first time. Biological screening revealed that several isolated sesquiterpenoids exhibited significant anti‐inflammatory activity with low IC_50_ values and minimal cytotoxicity, representing promising candidates for further pharmacological research. A focused mechanistic investigation on two structurally distinct compounds, **4** and **8**, revealed that both attenuated the LPS‐induced inflammatory response in macrophages by suppressing the secretion of pro‐inflammatory cytokines (IL‑6, IL‑1β) and downregulating the expression of key mediators (iNOS, COX‑2). A critical mechanistic divergence in upstream signaling was observed, as compound **8** significantly inhibited MAPK pathway activation (JNK, p38, and ERK1/2), while compound **4** showed no such effect. These finding highlights how structural differences in sesquiterpenoids can translate into distinct modes of action despite shared phenotypic outcomes. Collectively, this study makes a significant contribution to the development of phytochemistry and pharmacognosy in Kazakhstan, and highlights the potential of the local flora as a valuable source of bioactive compounds for the pharmaceutical industry.

## 4 Experimental Section

### 4.1 General Experimental Procedures

Optical rotations were recorded at ambient temperature on an Autopol VI automatic polarimeter (Rudolph), with concentrations reported in g/100 mL. ECD and UV spectra were obtained using a JASCO J‐815 spectrometer equipped with a 0.1 cm path‐length cell. IR spectra were measured on a Thermo Scientific Nicolet iS5 FT‐IR Spectrometer. ESIMS data were collected on a Thermo Fisher Finnigan LTQ Linear Ion Trap mass spectrometer or a Waters 2545 binary pump system with a Waters 2489 UV–vis detector (210 and 254 nm) and an Acquity QDa mass detector, while HRESIMS analyses were performed using an Agilent G6520 Q‐TOF mass spectrometer. NMR spectra were recorded on a Bruker Avance III 600, spectrometer with tetramethylsilane as the internal standard. Column chromatography (CC) was carried out on C_18_ reversed‐phase (RP‐18) silica gel (20–45 µm, Fuji Silysia Chemical Ltd.), D101 macroporous adsorption resin (Shanghai Hualing Resin Co. Ltd.), MCI gel (CHP20P, 75–150 µm, Mitsubishi Chemical Industries), Sephadex LH‐20 (GE Healthcare), and silica gel (200–300 and 300–400 mesh, Qingdao Haiyang Chemical Co. Ltd.). TLC analyses employed precoated silica gel GF254 plates (Qingdao Haiyang Chemical Co., Ltd.). Semi‐preparative HPLC was performed on a Waters 1525 binary pump system with a Waters 2489 detector (210 and 254 nm) using an X‐Bridge Prep C_18_ OBD column (10 mm × 250 mm, S‐5 µm). Preparative HPLC fractionation was performed using a Waters 2545 binary gradient module coupled with a Waters 2489 UV/vis detector (254 and 210 nm) and a Waters 2767 sample manager, with separations carried out on a Waters X‐Bridge Prep C18 column (250 × 19 mm, S‐5 µm, 130 Å). All the solvents used for CC were of analytical grade (Sinopharm Chemical Reagent Co., Ltd.), and solvents used for HPLC purification were of HPLC grade (J&K Scientific Ltd.).

### 4.2 Plant Material

The whole plants of *Taraxacum tianschanicum* Pavlov. were collected in April, 2021 from mountain of Trans‐Ili Alatau, the northernmost ridge of the Northern Tien Shan, Kazakhstan, at an altitude of 1500–1800 m above sea level, and the whole plants of *Artemisia gracilescens* Krasch. & Iljin. were collected in July, 2019 from Tarbagatai Mountains, Kazakhstan, at an altitude of 1200–1500 m above sea level. Janar Jenis was responsible for the collection and identification of the plant materials. Voucher specimens (TRTM‐2023‐1P, ARGS‐2023‐2P) are deposited at the Shanghai Institute of Materia Medica, Chinese Academy of Sciences.

### 4.3 Extraction and Isolation

The air‐dried and powdered whole plants of *T. tianschanicum* (4.0 kg) were soaked with 95% EtOH (30 L × 3) to give a residue (250.0 g), which was suspended in warm water followed by extraction with EtOAc. The EtOAc soluble extract (70.0 g) was subjected to CC (D101 macro porous absorption resin) eluted with 50%, 80%, and 95% EtOH in H_2_O to obtain four pooled fractions, 1–4. Fraction 2 (10.0 g) was divided into six parts (B1–B6) on an MCI gel column eluted with MeOH/H_2_O (30%–100%). Fraction B2 (3.0 g) was then fractionated on a column of silica gel (CH_2_Cl_2_/MeOH, 100:1 to 10:1) to produce five fractions (B2a–B2e). Fraction B2a (0.9 g) was chromatographed successively on RP‐C_18_ silica gel CC (MeOH/H_2_O, 40%–100%) and Sephadex LH‐20(MeOH), affording subfractions B2a1a–B2a1c. Then, through preparative HPLC (30% MeOH in H_2_O, 16 mL/min), compounds **1** (57.0 mg, *t*_R_ = 13.0 min) and **2** (5.0 mg, *t*_R_ = 22.8 min) were purified from fraction B2a1a (100.0 mg). From B2a1c (15.0 mg), compound **3** (5.6 mg, *t*_R_ = 15.3 min) was obtained by semi‐preparative HPLC (50 % MeOH in H_2_O, 3 mL/min).

Using the same procedure as described above, the air‐dried whole plants of *A. gracilescens* (5.0 kg) were macerated with 95% EtOH, and then extracted with EtOAc/H_2_O. The resulting EtOAc part (90.0) was subjected to MCI CC using a MeOH/H_2_O gradient (30%–100%), affording ten parts, A1–A10. Fraction A4 (15.0 g) was separated on silica gel CC with a petroleum ether/acetone gradient (15:1 to 1:5), yielding nine subfractions (A4a–A4i). Fraction A4b (5.0 g), initially a complex mixture, was left to stand in MeOH at room temperature, resulting in the formation of crystals and identified as compound **7** (2.0 g). Fraction A4c (500.0 mg) was chromatographed on silica gel CC with CHCl_3_/acetone gradient (100:1 to 10:1) as eluents to give four fractions (A4c1–A4c4), and the soluble part of fraction A4c2 (30.0 mg) was further purified by semi‐preparative HPLC (45 % CH_3_CN in H_2_O, 3 mL/min), affording compounds **4** (2.0 mg, *t*_R_ = 8.7 min) and **6** (1.2 mg, *t*_R_ = 20.2 min). Fraction A4c3 (150.0 mg) was fractioned by silica gel CC using a stepwise gradient of petroleum ether/CHCl_3_ (10:1 to 0:1), followed by CH_2_Cl_2_/MeOH (100:1 to 10:1), and the resulting fraction A4c3b (40.0 mg) was purified by semi‐preparative HPLC (50 % CH_3_CN in H_2_O, 3 mL/min) to provide compounds **5** (2.0 mg, *t*_R_ = 13.5 min) and **9** (1.5 mg, *t*_R_ = 11.3 min). Fraction A4d (400.0 mg) was chromatographed by a silica gel column with petroleum ether/CHCl_3_ (10:1 to 0:1) and then CHCl_3_/EtOAc (50:1 to 1:1) as the eluents to give nine fractions (A4d1–A4d9), and A4d2 (20.0 mg) was purified by semi‐preparative HPLC (52 % CH_3_CN in H_2_O, 3 mL/min) to afford compound **8** (2.0 mg, *t*_R_ = 14.0 min). Similarly, fraction A4e (15.0 mg) was subjected to silica gel fractionation followed by semi‐preparative HPLC (55% CH_3_CN in H_2_O, 3 mL/min) to obtain compound **10** (1.0 mg, *t*_R_ = 6.8 min).

### 4.4 (11R)‐11,13‐Dihydrotaraxinic Acid (**1**)

Light yellow gum; ${\left[\alpha \right]}_{D}^{20}$ −53.5 (*c* 0.2, MeOH); UV(MeOH) * λ*_max_ (log ε) 207 (0.60), 240 (0.19) nm; ECD (MeOH) *λ* (Δ*ε*) 239 (5.6), 220 (44), 215 (−14) nm; IR (KBr) *ν*_max_ 3444, 2934, 2875, 1767, 1704, 1456, 1384, 1180, 1132, 966 cm^−1^; ^1^H and ^13^C NMR data (CDCl_3_), see Table 1; (+)‐ESIMS *m/z* 265.08 [M + H]^+^, (−)‐ESIMS *m/z* 263.04 [M ‒ H]^‒^; (−)­HRESIMS *m/z* 263.1288 [M ‒ H]^‒^ (calcd for C_15_H_19_O_4_^‒^, 263.1289).

### 4.5 ECD Calculations

Conformational search, geometric optimization, and frequency calculations results of compound **1** were performed according to previously published methods [24, 25]. Conformational screening was carried out using the Monte Carlo multiple minimum (MCMM) method with the OPLS‐2005 force field in the Macromodel 10.2 program (Schrödinger Release 2015‐2: MacroModel, Schrödinger, LLC, New York, USA). An energy window of 3.01 kcal/mol was used in the calculations (Table S3). ECD spectra were calculated using the TDDFT method PCM/ωB97XD/6‐311G** in methanol. Boltzmann averaging of the spectra was performed in SpecDis 1.71 using Gaussian functions. The final calculated ECD spectra were adjusted using σ values and UV spectrum corrections, which were 0.30 eV and 9 nm for compound **1**.

### 4.6 Biological Evaluation

#### 4.6.1 Materials and Cell Culture

Dulbecco's Modified Eagle Medium (DMEM) and Ham's F‐12K medium (F‐12K) were purchased from Gibco (Beijing, China), fetal bovine serum (FBS) was obtained from Zeta Life (NJ, USA). 3‐[4,5‐Dimethylthiazole‐2‐yl]‐2,5‐diphenyltetrazolium bromide (MTT) and lipopolysaccharide (LPS) were purchased from Solarbio (Beijing, China), and Cell Counting Kit‐8 (CCK‐8) was purchased from APExBIO (Houston, USA).

The murine macrophage cell line RAW 264.7 (RRID:CVCL_0493) and three human tumor cell lines, including breast adenocarcinoma cell line MDA‐MB‐231 (RRID: CVCL_0062), lung adenocarcinoma cell line A549 (RRID: CVCL_0023), and hepatocellular carcinoma cell line HepG2 (RRID: CVCL_0027), were purchased from Procell Life Science&Technology Co., Ltd. (Wuhan, China). All cell lines were authenticated by the supplier using short tandem repeat (STR) profiling and were tested negative for mycoplasma contamination.

Griess reagent was obtained from Sigma (St. Louis, Missouri). The RIPA lysis buffer, PMSF, and BCA protein determination kit were obtained from Beyotime Biotechnology Co. Ltd. (Shanghai, China). ELISA kits for mice (IL‐6, IL‐1β) were purchased from Abisin (Beijing, China). Antibodies: iNOS (#13120), COX‐2 (#12282), MAPK pathway sampler kit (#9926), phospho‐MAPK pathway sampler kit (#9910) and GAPDH (#2118) were all obtained from Cell signaling Technology (Beverly, MA, USA).

#### 4.6.2 Cytotoxicity Assays Against Tumor Cells

Compounds **1–5**, **7**, and **8** were subjected to cytotoxic evaluation against three cancer cell lines (MDA‐MB‐231, A549, and Hep G2) using established protocols [27, 28, 29]. Briefly, tumor cells were seeded into 96‐well plates (10^4^ cells/well) and allowed to adhere for 24 h. Then, the cells were treated with each test compound dissolved in DMSO (20 *µ*M). Doxorubicin was used as the positive control, exhibiting IC_50_ values of 3.31 ± 0.2, 1.48 ± 0.21, and 1.58 ± 0.01 µM against MDA‐MB‐231, A549, and Hep G2 cells, respectively. After continuous incubation for 48 h, 20 µL MTT solution (5 mg/mL) was added to per well and incubated at 37°C for 4 h. After removing the medium, 150 µL DMSO was added into each well, and the absorption at 570 nm was measured using SpectraMax iD3 Platform (Molecular Devices, USA). The experiment was carried out three times.

#### 4.6.3 Anti‐Inflammatory Assay

The anti‐inflammatory assay including nitric oxide (NO) production and cell viability assays, were carried out according to previously reported protocol [30, 31, 32, 33]. Briefly, RAW 264.7 macrophages were seeded into 96‐well plates (3 × 10^4^ cells/well) and cultured for 24 h. Then, the test compounds (2 µL/well) and lipopolysaccharide (LPS, 0.05 µg/mL) were added. After 24 h of incubation, 70 µL of the medium was transferred to a new plate and mixed with 70 µL of the Griess reagent, and the absorbance was measured at 546 nm to determine NO levels. Subsequently, 70 µL of fresh DMEM medium was added to replenish each well of the original 96‐well plate, and 10 µL of CCK‐8 solution was added to each well. After incubation at 37°C for 2 h, the absorbance was detected at a wavelength of 450 nm to get cell viability data. Data analysis was performed using nonlinear regression curve fitting with GraphPad Prism 8.0 to determine IC_50_ values.

#### 4.6.4 Measurement of Cytokines

The ELISA kits were used to measure the contents of IL‐6 and IL‐1β following the manufacturer's instructions. RAW 264.7 cells were seeded in 12‐well plates, in the presence of indicated concentration of compounds, and were stimulated with LPS (0.5 µg/mL) for 12 h [31, 33]. After this, the culture medium was collected to perform the ELISA assays. The absorbance intensity at 450 nm was detected using a microplate reader. The experiment was performed in triplicate and the cytokine level was calculated using the standard curve.

#### 4.6.5 Western Blot Analysis

RAW 264.7 cells were seeded in 6‐well plates, in the presence of indicated concentration of compounds, and were stimulated with LPS (0.5 µg/mL) for 12 h [32]. Then cells were collected and washed twice with PBS, then lysed with RIPA lysis buffer and centrifuged to obtain total protein. The protein concentration was quantified using the BCA protein determination kit. Equal amounts of protein were subjected to 10% SDS‐PAGE and then transferred onto PVDF membranes. Membranes were blocked with 5% skimmed milk and followed by overnight incubation with primary antibodies at 4°C and a 2 h incubation with HRP‐conjugated secondary antibodies at room temperature. Finally, the protein bands were visualized using enhanced chemiluminescence apparatus. Band density was quantified using ImageJ software.

#### 4.6.6 Statistical Analysis

GraphPad Prism 8.0 software (San Diego, CA, USA) was used to analyze the biological experimental data. The data are presented as mean ± SD from three independent biological replicates (*n* = 3), each performed with technical triplicates. Statistical significance was determined using one‐way analysis of variance (ANOVA) followed by Dunnett's post‐hoc test for multiple comparisons against the LPS group. A *p*‐value of < 0.05 was considered statistically significant. The exact sample size (*n*) and statistical method used for each experiment are also indicated in the corresponding figure legends.

## Author Contributions

A.Z. conducted the isolation and structural elucidation of the compounds and contributed to manuscript writing. W.‐J.G. designed and performed the biological activity assays and participated in manuscript preparation. J.J. was responsible for the collection and identification of the plant materials. C.‐H.F. and P.‐Q.W.carried out the data analysis and interpretation. J.‐M.Y. and B.Z. conceived and supervised the project, guided the research, and reviewed and edited the manuscript. All authors have read and agreed to the published version of the manuscript.

## Conflicts of Interest

The authors declare no conflicts of interest.

## Supporting information




**Supporting File**: cbdv71683‐sup‐0001‐SuppMat.pdf.

## Data Availability

The data that support the findings of this study are available in the supplementary material of this article.

## References

[1] L. Grudzinskaya , N. Gemejiyeva , and Z. Z. Karzhaubekova , “The Kazakhstan Medicinal Flora Survey in a Leading Families Volume,” Fundamental and Experimental Biology 100, no. 4 (2020): 39–51, 10.31489/2020bmg4/39-51.

[2] Z. B. Ashirova , Z. Z. Kuzhantaeva , Z. T. Abdrassulova , G. Z. Shaimerdenova , and G. K. Atanbaeva , “Studying Phytochemical Features of Three Asteraceae Herbs Growing Wild in Kazakhstan,” Floresta E Ambiente 28, no. 4 (2021): e20210060, 10.1590/2179-8087-FLORAM-2021-0060.

[3] S. Adekenov , “Sesquiterpene Lactones From Plants of the Family Asteraceae in the Kazakhstan Flora and Their Biological Activity,” Chemistry of Natural Compounds 31, no. 1 (1995): 21–25, 10.1007/BF01167564.

[4] K. Schütz , R. Carle , and A. Schieber , “ *Taraxacum*—A Review on Its Phytochemical and Pharmacological Profile,” Journal of Ethnopharmacology 107, no. 3 (2006): 313–323, 10.1016/j.jep.2006.07.021. 16950583

[5] C. Hu and D. D. Kitts , “Antioxidant, Prooxidant, and Cytotoxic Activities of Solvent‐Fractionated Dandelion (*Taraxacum officinale*) Flower Extracts In Vitro,” Journal of Agricultural and Food Chemistry 51, no. 1 (2003): 301–310, 10.1021/jf0258858. 12502425

[6] T. E. Wallaart , N. Pras , and W. J. Quax , “Isolation and Identification of Dihydroartemisinic Acid Hydroperoxide from *Artemisia annua*: A Novel Biosynthetic Precursor of Artemisinin,” Journal of Natural Products 62, no. 8 (1999): 1160–1162, 10.1021/np9900122. 10479327

[7] G. D. Brown , “The Biosynthesis of Artemisinin (Qinghaosu) and the Phytochemistry of *Artemisia annua* L. (Qinghao),” Molecules 15, no. 11 (2010): 7603–7698, 10.3390/molecules15117603. PMC6259225 21030913

[8] H. Tantau , C. Balko , B. Brettschneider , G. Melz , and K. Dörffling , “Improved Frost Tolerance and Winter Survival in Winter Barley (*Hordeum vulgare* L.) by In Vitro Selection of Proline Over‐Accumulating Lines,” Euphytica 139, no. 1 (2004): 19–32, 10.1007/s10681-004-2231-2.

[9] Y. Song , J. J. John Martin , X. Liu , et al., “Unraveling the Response of Secondary Metabolites to Cold Tolerance in Oil Palm by Integration of Physiology and Metabolomic Analyses,” BMC Plant Biology 25, no. 1 (2025): 279, 10.1186/s12870-025-06292-5. PMC11877684 40033206

[10] S. Liu , T. Li , S. Fang , et al., “Metabolic Profiling and Gene Expression Analyses Provide Insights into Cold Adaptation of an Antarctic Moss *Pohlia nutans* ,” Frontiers in Plant Science 13 (2022): 1006991, 10.3389/fpls.2022.1006991. PMC9514047 36176693

[11] J. Liu , N. Zhang , and M. Liu , “A New Inositol Triester from *Taraxacum mongolicum* ,” Natural Product Research 28, no. 7 (2014): 420–423, 10.1080/14786419.2013.867443. 24432736

[12] K. Michalska , J. Marciniuk , and W. Kisiel , “Sesquiterpenoids and Phenolics From Roots of *Taraxacum udum* ,” Fitoterapia 81, no. 5 (2010): 434–436, 10.1016/j.fitote.2009.12.004. 20006977

[13] Y.‐P. Chen , S.‐L. Wang , Y.‐H. Shen , and Z.‐J. Wu , “Chemical Constituents From *Ainsliaea glabra* ,” Guihaia 34, no. 3 (2014): 402–407, 10.3969/j.issn.1000-3142.2014.03.022.

[14] D. E. Dusmatova , K. M. Bobakulov , R. F. Mukhamatkhanova , et al., “Isolation of Cytotoxic Sesquiterpene Lactones From the *Tanacetopsis karataviensis* (Kovalevsk.) Kovalevsk,” Natural Product Research 35, no. 12 (2021): 1939–1948, 10.1080/14786419.2019.1647423. 31359772

[15] C. J. Bethencourt‐Estrella , N. Nocchi , A. López‐Arencibia , et al., “Antikinetoplastid Activity of Sesquiterpenes Isolated From the Zoanthid *Palythoa Aff. clavata* ,” Pharmaceuticals 14, no. 11 (2021): 1095, 10.3390/ph14111095. PMC8625207 34832876

[16] M. Lazanaki , G. Tsikalas , O. S. Tsiftsoglou , et al., “Secondary Metabolites and Their Biological Evaluation from the Aerial Parts of *Staehelina Uniflosculosa S*ibth. & Sm. (Asteraceae),” International Journal of Molecular Sciences 25, no. 19 (2024): 10586, 10.3390/ijms251910586. PMC11476517 39408914

[17] J.‐L. Wang , “Sesquiterpenoids From *Artemisia integrifolia* ,” Zhongcaoyao (2018): 3758–3762, 10.7501/j.issn.0253-2670.2018.16.005.

[18] A. Natarajan , C. Tsai , S. I. Khan , et al., “The Photoarrangement of *α*‐Santonin is a Single‐Crystal‐to‐Single‐Crystal Reaction: A Long Kept Secret in Solid‐State Organic Chemistry Revealed,” Journal of the American Chemical Society 129, no. 32 (2007): 9846–9847, 10.1021/ja073189o. 17645337

[19] G.‐X. Fan , L.‐L. Dong , H.‐H. Li , et al., “Sesquiterpenoids and Other Chemical Components from the Roots of *Dolomiaea souliei* ,” Chemistry of Natural Compounds 52, no. 4 (2016): 754–757, 10.1007/s10600-016-1766-5.

[20] C.‐M. Sun , W.‐J. Syu , M.‐J. Don , J.‐J. Lu , and G.‐H. Lee , “Cytotoxic Sesquiterpene Lactones From the Root of *Saussurea lappa* ,” Journal of Natural Products 66, no. 9 (2003): 1175–1180, 10.1021/np030147e. 14510592

[21] L.‐F. Ding , J. Su , Z.‐H. Pan , et al., “Cytotoxic Sesquiterpenoids From the Leaves of *Magnolia grandiflora* ,” Phytochemistry 155 (2018): 182–190, 10.1016/j.phytochem.2018.08.006. 30145456

[22] X. H. Meng , H. Lv , X. Q. Ding , et al., “Sesquiterpene Lactones With Anti‐Inflammatory and Cytotoxic Activities From the Roots of *Cichorium intybus* ,” Phytochemistry 203 (2022): 113377, 10.1016/j.phytochem.2022.113377. 35988742

[23] M. Cui , X. Wang , T. Yu , X. M. Liu , and X. M. Zhang , “Unveiling the Genus *Taraxacum*: From Folk Medicine to Chemodiversity‐Driven Pharmacological and Toxicological Outcomes—A Systematic Review,” Journal of Ethnopharmacology 360 (2026): 121231, 10.1016/j.jep.2026.121231. 41554454

[24] Z. Z. Zhumagaliyeva , R. I. Dzhalmakhanbetova , S. K. Eleupaeva , and V. I. Korchyn , “Antioxidant Activity of Aminоderivatives of Santonin Extracted From the Plant *Artemisia* Gracil. Krasch,” Fundamental and Experimental Biology 90, no. 2 (2018): 40–45, 10.31489/2018bmg2/40-45.

[25] R. M. Lucas , L. Luo , and J. L. Stow , “ERK1/2 in Immune Signalling,” Biochemical Society Transactions 50, no. 5 (2022): 1341–1352, 10.1042/Bst20220271. PMC9704528 36281999

[26] J. S. C. Arthur and S. C. Ley , “Mitogen‐Activated Protein Kinases in Innate Immunity,” Nature Reviews Immunology 13, no. 9 (2013): 679–692, 10.1038/nri3495. 23954936

[27] P.‐Q. Wu , C.‐H. Fang , B. Zhou , and J.‐M. Yue , “Integrated Strategy by Combining Preparative HSCCC With HPLC–UV–QDa for Efficient Isolation of Specific Friedelane‐Type Triterpenoids from *Salix tetrasperma* ,” Journal of Chromatography A 1753 (2025): 465912, 10.1016/j.chroma.2025.465912. 40315770

[28] Q. Zhu , Y. Li , C. Wang , et al., “Cytotoxic Diterpenoids From *Croton kongensis* Inhibiting Tumor Proliferation and Migration,” Bioorganic Chemistry 152 (2024): 107739, 10.1016/j.bioorg.2024.107739. 39186915

[29] P.‐P. An , H. Huang , S.‐J. Ru , et al., “Intriguing Steroid Glycosides for Cancer Therapy by Suppressing the DNA Damage Response and mTOR/S6K Signaling Pathways,” Bioorganic Chemistry 151 (2024): 107619, 10.1016/j.bioorg.2024.107619. 39024806

[30] Y. Gao , Y. Li , J.‐S. Zhou , et al., “Harnessing Functional Food Sources: Deriving Anti‐Inflammatory and Antibacterial Naphthalene Derivatives From the Edible Bulbs of *Eleutherine bulbosa* ,” Journal of Agricultural and Food Chemistry 73, no. 7 (2025): 4126–4136, 10.1021/acs.jafc.4c12108. 39930623

[31] Y. Mi , Y.‐H. Ren , Y. Li , et al., “Rare Chlorinated C19 Steroids Hocarnoids A and B From *Hoya carnosa*: Isolation, Semi‐Synthesis, and Anti‐Inflammation,” The Journal of Organic Chemistry 90, no. 13 (2025): 4714–4724, 10.1021/acs.joc.5c00211. 40111232

[32] Y. Gao , Y. Li , J.‐S. Zhou , et al., “Eleutherlenes A–D, Diverse Types of Naphthalene Derivatives With Anti‐Inflammatory Activity From *Eleutherine bulbosa* ,” Organic Letters 27, no. 4 (2025): 1066–1071, 10.1021/acs.orglett.4c04798. 39829017

[33] P.‐Q. Wu , Y. Li , Y.‐H. Ren , et al., “Anti‐inflammatory Salicin Derivatives From the Barks of *Salix tetrasperma* ,” Journal of Agricultural and Food Chemistry 72, no. 16 (2024): 9215–9226, 10.1021/acs.jafc.4c01061. 38602386

