# Supplementary material for: Anti‑Inflammatory Sesquiterpenoids From Two Asteraceae Plants Native to Kazakhstan: Isolation, Structural Elucidation, and Mechanistic Insights
Source: Chem Biodivers. 2026 Sep 20;23(9):e71683. doi: 10.1002/cbdv.71683 (PMC13590133; doi:10.1002/cbdv.71683)
Supplement: Supplementary file 1 [file CBDV-23-e71683-s001.pdf]

Supporting Information for

Anti-inflammatory Sesquiterpenoids from Two Asteraceae Plants Native to Kazakhstan: Isolation, Structural Elucidation, and Mechanistic Insights

Arailym Zhomartova,[1a,b,c] Wen-Jing Gao,[1d] Janar Jenis,[c] Chu-Hong Fang,[d] Pei-Qian Wu,[a] Jian-Min Yue,*[a,b,d] and Bin Zhou*[a,b]

[a] Ethnomedicine and Biofunctional Molecule Research Center, State Key Laboratory of Drug Research, Shanghai Institute of Materia Medica, Chinese Academy of Sciences, 555 Zuchongzhi Road, Shanghai 201203, China (E-mail: jmyue@simm.ac.cn, zbbrush@simm.ac.cn)

[b] University of Chinese Academy of Sciences, No. 19A Yuquan Road, Beijing 100049, China

[c] The Research Center for Medicinal Plants, Al-Farabi Kazakh National University, al-Farabi Ave. 71, Almaty 050040, Kazakhstan

[d] Shandong Laboratory of Yantai Drug Discovery, Bohai Rim Advanced Research Institute for Drug Discovery, Yantai, Shandong 264117, Republic of China

*Corresponding authors: jmyue@simm.ac.cn (J.-M. Yue), zbbrush@simm.ac.cn (B. Zhou).

[1] The authors contributed equally to the work.

Contents

1. Information about identified compounds S1
Table S1. Information About Compounds. S1
2. Anti-inflammatory activity S2
Table S2. Anti-Inflammatory Activity of Selected Compounds. S2
3. 1D and 2D NMR, and MS data of Compounds 1–10 .... S3
Fig. S1. ^{1}H NMR spectrum of compound **1** in $CDCl_3$ S3
Fig. S2. ^{13}C NMR spectrum of compound **1** in $CDCl_3$ S3
Fig. S3. HSQC spectrum of compound **1** in $CDCl_3$ S4
Fig. S4. HMBC spectrum of compound **1** in $CDCl_3$ S4
Fig. S5. ^{1}H–^{1}H COSY spectrum of compound **1** in $CDCl_3$ S5
Fig. S6. NOESY spectrum of compound **1** in $CDCl_3$ S5
Fig. S7. (±)-ESIMS spectra of compound **1** S6
Fig. S8. (+)-HRESIMS spectrum of compound **1** S6
Fig. S9. IR spectrum of compound **1** S7
Fig. S10. ^{1}H NMR spectrum of compound **2** in $CDCl_3$ S7
Fig. S11. ^{1}H NMR spectrum of compound **2** in methanol-d_4 .... S8
Fig. S12. ^{13}C NMR spectrum of compound **2** in methanol-d_4 .... S8
Fig. S13. (±)-ESIMS spectra of compound **2** S9
Fig. S14. ^{1}H NMR spectrum of compound **3** in $CDCl_3$ S9
Fig. S15. (±)-ESIMS spectra of compound **3** S10
Fig. S16. ^{1}H NMR spectrum of compound **4** in methanol-d_4 .... S10
Fig. S17. ^{13}C NMR spectrum of compound **4** in methanol-d_4 .... S11
Fig. S18. (±)-ESIMS spectra of compound **4** S11
Fig. S19. ^{1}H NMR spectrum of compound **5** in DMSO-d_6 .... S12
Fig. S20. ^{13}C NMR spectrum of compound **5** in DMSO-d_6 .... S12
Fig. S21. (±)-ESIMS spectra of compound **5** S13
Fig. S22. ^{1}H NMR spectrum of compound **6** in methanol-d_4 .... S13
Fig. S23. ^{13}C NMR spectrum of compound **6** in methanol-d_4 .... S14
Fig. S24. (±)-ESIMS spectra of compound **6** S14
Fig. S25. ^{1}H NMR spectrum of compound **7** in $CDCl_3$ S15
Fig. S26. ^{13}C NMR spectrum of compound **7** in $CDCl_3$ S15
Fig. S27. (±)-ESIMS spectra of compound **7** S16
Fig. S28. ^{1}H NMR spectrum of compound **8** in $CDCl_3$ S16
Fig. S29. ^{13}C NMR spectrum of compound **8** in $CDCl_3$ S17
Fig. S30. (±)-ESIMS spectra of compound **8** S17

Fig. S31. ^{1}H NMR spectrum of compound **9** in methanol-d_4 .... S18
Fig. S32. ^{13}C NMR spectrum of compound **9** in methanol-d_4 .... S18
Fig. S33. (±)-ESIMS spectra of compound **9** S19
Fig. S34. ^{1}H NMR spectrum of compound **10** in $CDCl_3$ S19
Fig. S35. ^{13}C NMR spectrum of compound **10** in $CDCl_3$ S20
Fig. S36. (±)-ESIMS spectra of compound **10** S20
4. Computational Section S21
Table S3. PCM/ωB97XD/6-311G** reoptimized conformers of compound **1**. S21

1. Information about identified compounds

Table S1. Information About Compounds.

Name	Structure	Reference	Plant
Luteolin	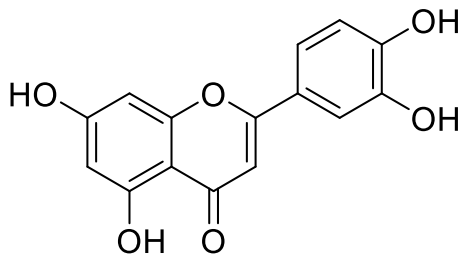	L.-C. Lin, Y.-F. Pai and T.-H. Tsai. Isolation of luteolin and luteolin-7-*O*-glucoside from *Dendranthema morifolium* Ramat Tzvel and their pharmacokinetics in rats. *J. Agric. Food Chem.* **63**, (35), 7700–7706 (2015). https://pubs.acs.org/doi/10.1021/jf505848z	*Taraxacum tianschanicum*
Isoetin-7-*O*-*β*-D-glucopyranosyl-2′-*O*-*α*-L-arabinopyranoside	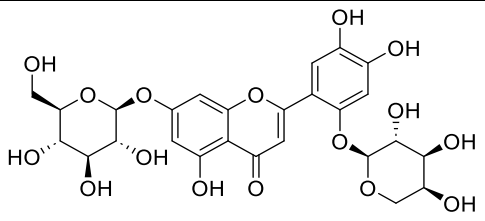	S. Y. Shi, Q. Zhou, H. Peng, C. X. Zhou, M. H. Hu, Q. F. Tao, X. J. Hao, J. Stöckigt and Y. Zhao. Four new constituents from *Taraxacum mongolicum*. *Chin. Chem. Lett.* **18**, (11), 1367–1370 (2007). https://doi.org/10.1016/j.cclet.2007.09.005	*Taraxacum tianschanicum*
Genistein	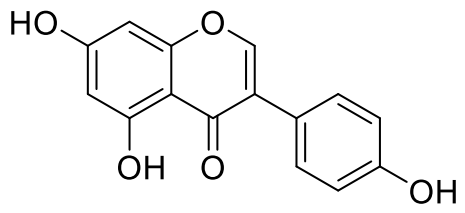	A. Tadjong Tcho, J. L. Djouaka Bavoua, A. Ngouonpe Wembe, E. G. Gompe Bobda, F. Majoumo, J. M. Leuche, E. Ndonfack Tiofack, J. A. Mbah and F. A. A. Toze. New flavanone and other constituents from *Erythrina senegalensis* A. DC. (Fabaceae). *Nat. Prod. Res.* **39**, (20), 5967–5977 (2025). https://doi.org/10.1080/14786419.2024.2364258	*Taraxacum tianschanicum*
Quercetin	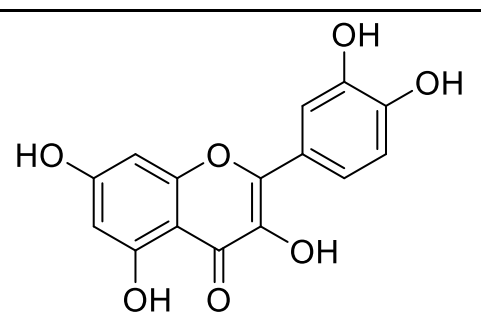	A. Adhikari-Devkota, S. M. I. Elbashir, T. Watanabe and H. P. Devkota. Chemical constituents from the flowers of *Satsuma mandarin* and their free radical scavenging and *α*-glucosidase inhibitory activities. *Nat. Prod. Res.* **33**, (11), 1670–1673 (2019). https://doi.org/10.1080/14786419.2018.1425856	*Taraxacum tianschanicum*
Luteolin 7-*O*-glucoside	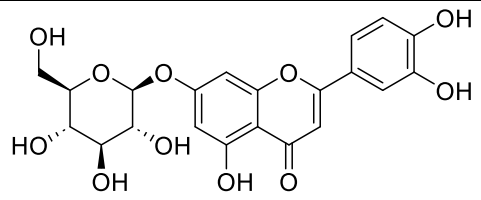	L.-C. Lin, Y.-F. Pai and T.-H. Tsai. Isolation of luteolin and luteolin-7-*O*-glucoside from *Dendranthema morifolium* Ramat Tzvel and their pharmacokinetics in rats. *J. Agric. Food Chem.* **63**, (35), 7700–7706 (2015). https://pubs.acs.org/doi/10.1021/jf505848z	*Taraxacum tianschanicum*
1-hexadecanoyl propan-2.3-diol	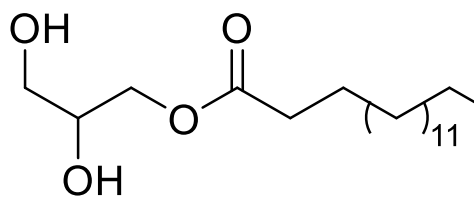	Z. Y. C. Guang Jin Pan, Hans R. Schöler & Duanqing Pei. Stem cell pluripotency and transcription factor. *Cell Res.* (2002). https://doi.org/0939-5075/2000/0700-0500	*Taraxacum tianschanicum*
5,7,3′-trihydroxy-6,4′,5′-trimethoxyflavone	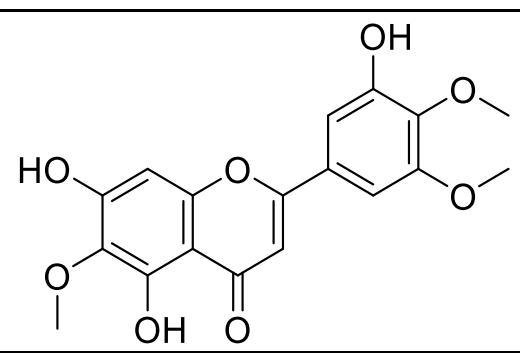	J. A. Marco, O. Barberá, S. Rodríguez, C. Domingo and J. Adell. Flavonoids and other phenolics from *Artemisia hispanica*. *Phytochemistry*. **27**, (10), 3155–3159 (1988). https://doi.org/10.1016/0031-9422(88)80018-9	*Artemisia gracilescens*
3(ζ)-hydroxy-octadeca-4(*E*),6(*Z*),15(*Z*)-trienoic acid[a]	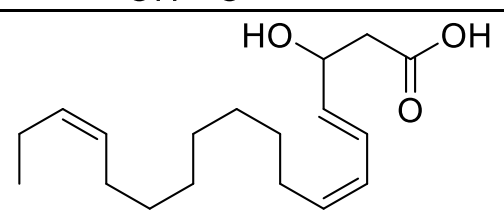	R.-W. Jiang, M. E. Hay, C. R. Fairchild, J. Prudhomme, K. Le Roch, W. Aalbersberg and J. Kubanek. Antineoplastic unsaturated fatty acids from *Fijian macroalgae*. *Phytochemistry*. **69**, (13), 2495–2500 (2008). https://doi.org/10.1016/j.phytochem.2008.07.005	*Artemisia gracilescens*

[a]Data suggest that the structure in this context may be incomplete or require refinement.

2. Anti-inflammatory activity

Table S2. Anti-Inflammatory Activity of Selected Compounds.

Compound	Inhibition (%)	IC_{50} (μM)
**1**	21.81	>20
**2**	44.31	>20
**3**	77.79	8.37 ± 0.99
**4**	99.04	1.15 ± 0.05
**5**	99.04	1.52 ± 0.16
**7**	86.13	>20
**8**	98.03	1.88 ± 0.17
**Dexamethasonen**		0.040 ± 0.004

3. 1D and 2D NMR, and MS data of Compounds 1–10

Fig. S1. ^{1}H NMR spectrum of compound **1** in $CDCl_3$

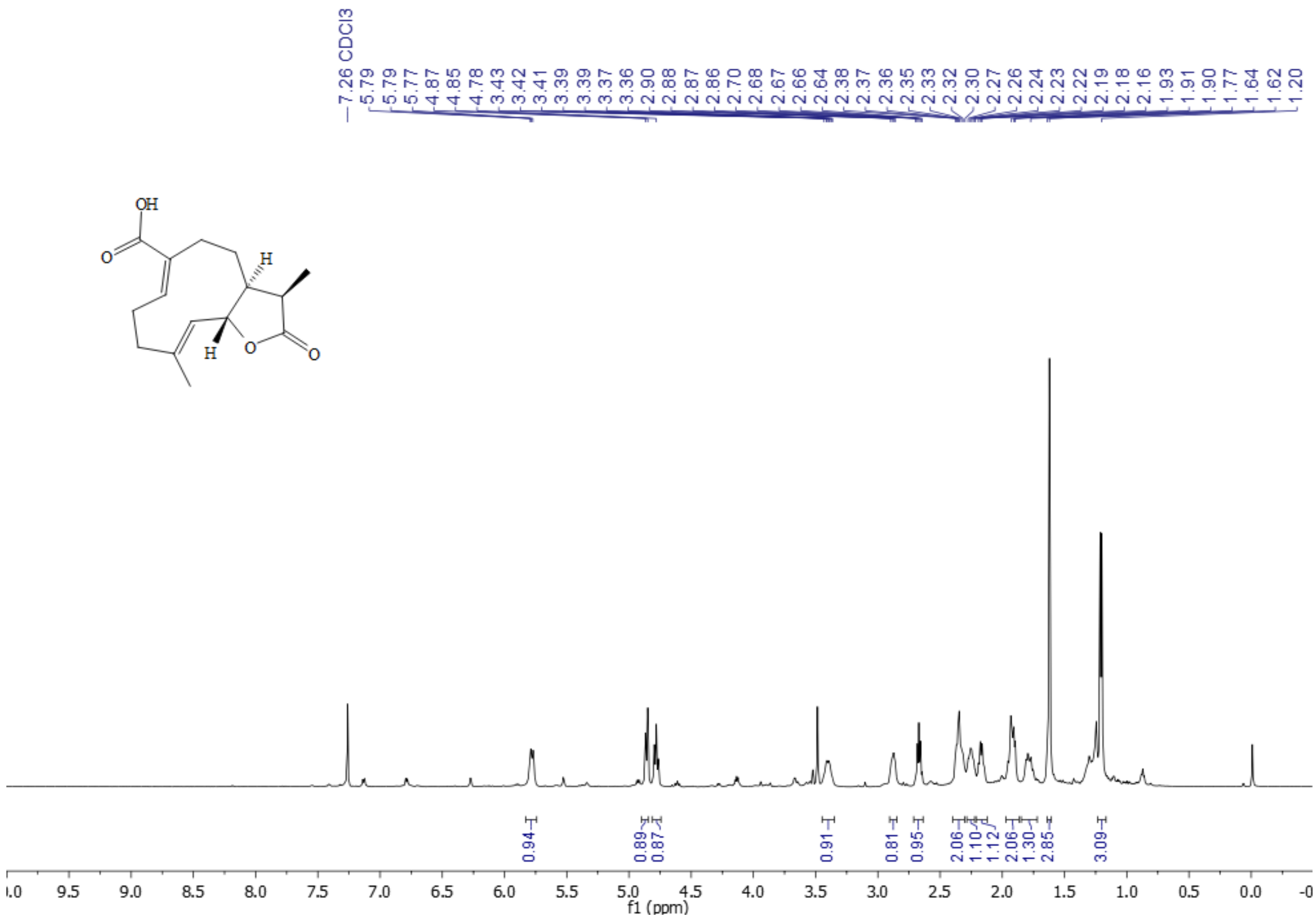

Fig. S2. ^{13}C NMR spectrum of compound **1** in $CDCl_3$

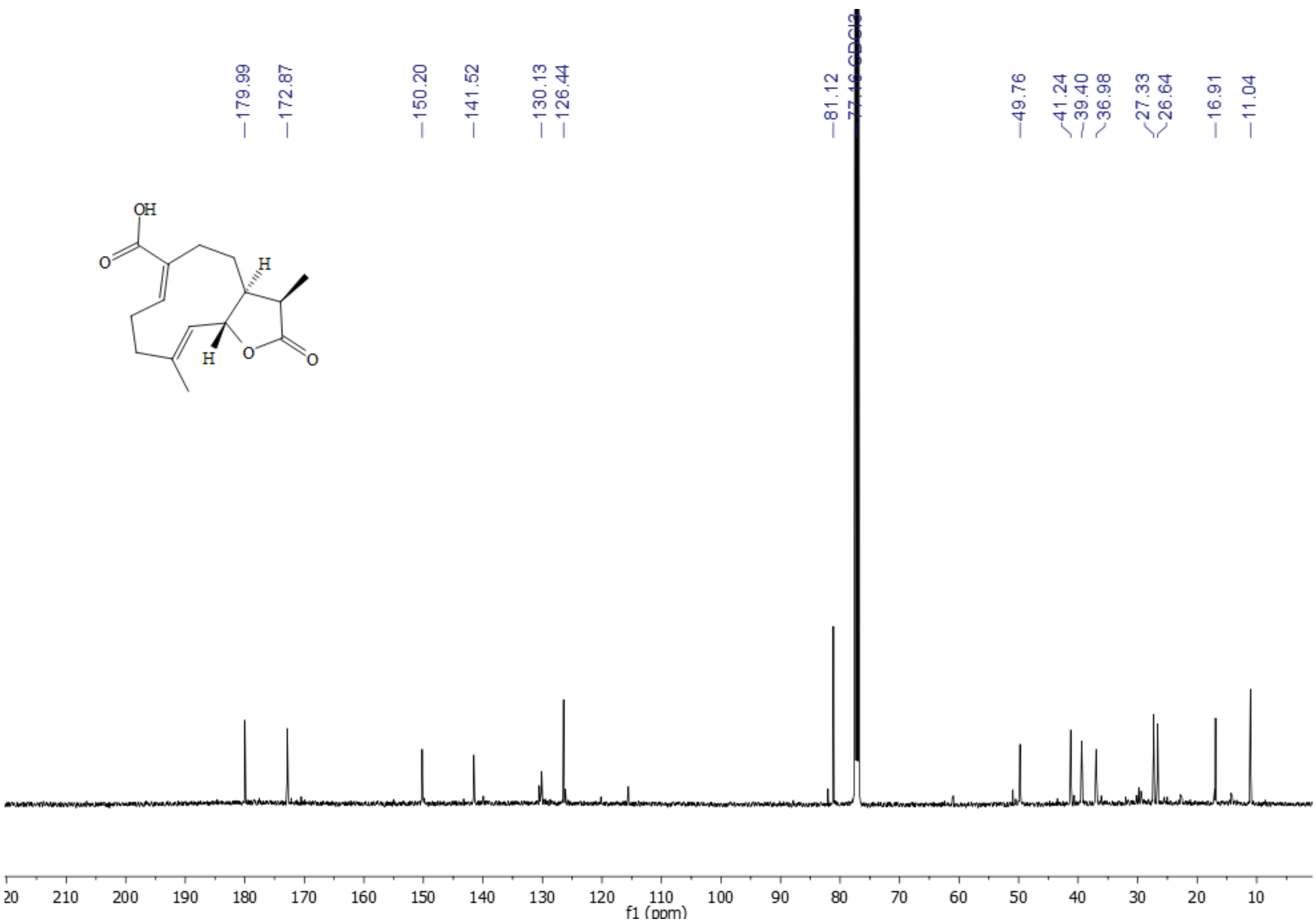

Fig. S3. HSQC spectrum of compound **1** in $CDCl_3$

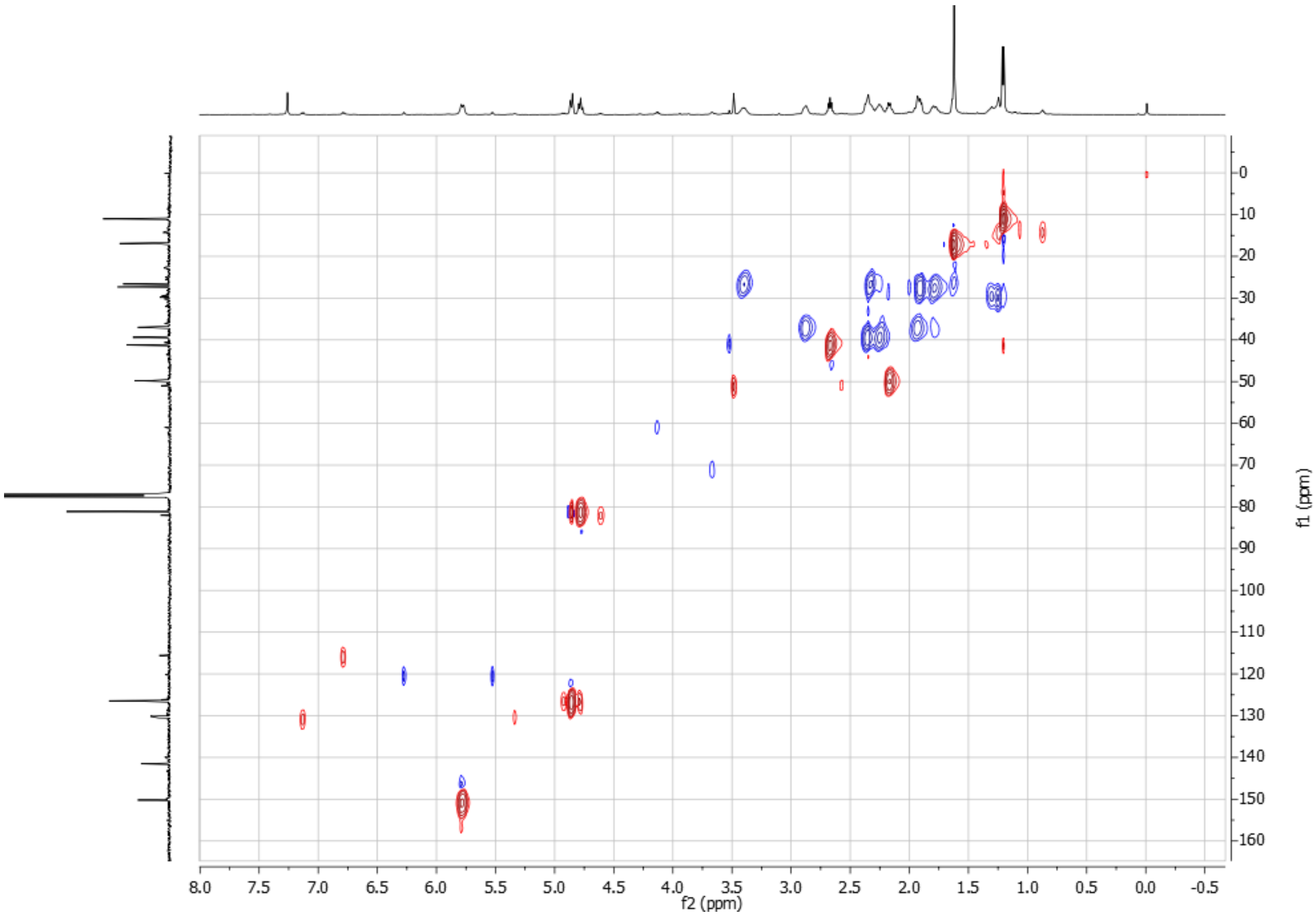

Fig. S4. HMBC spectrum of compound **1** in $CDCl_3$

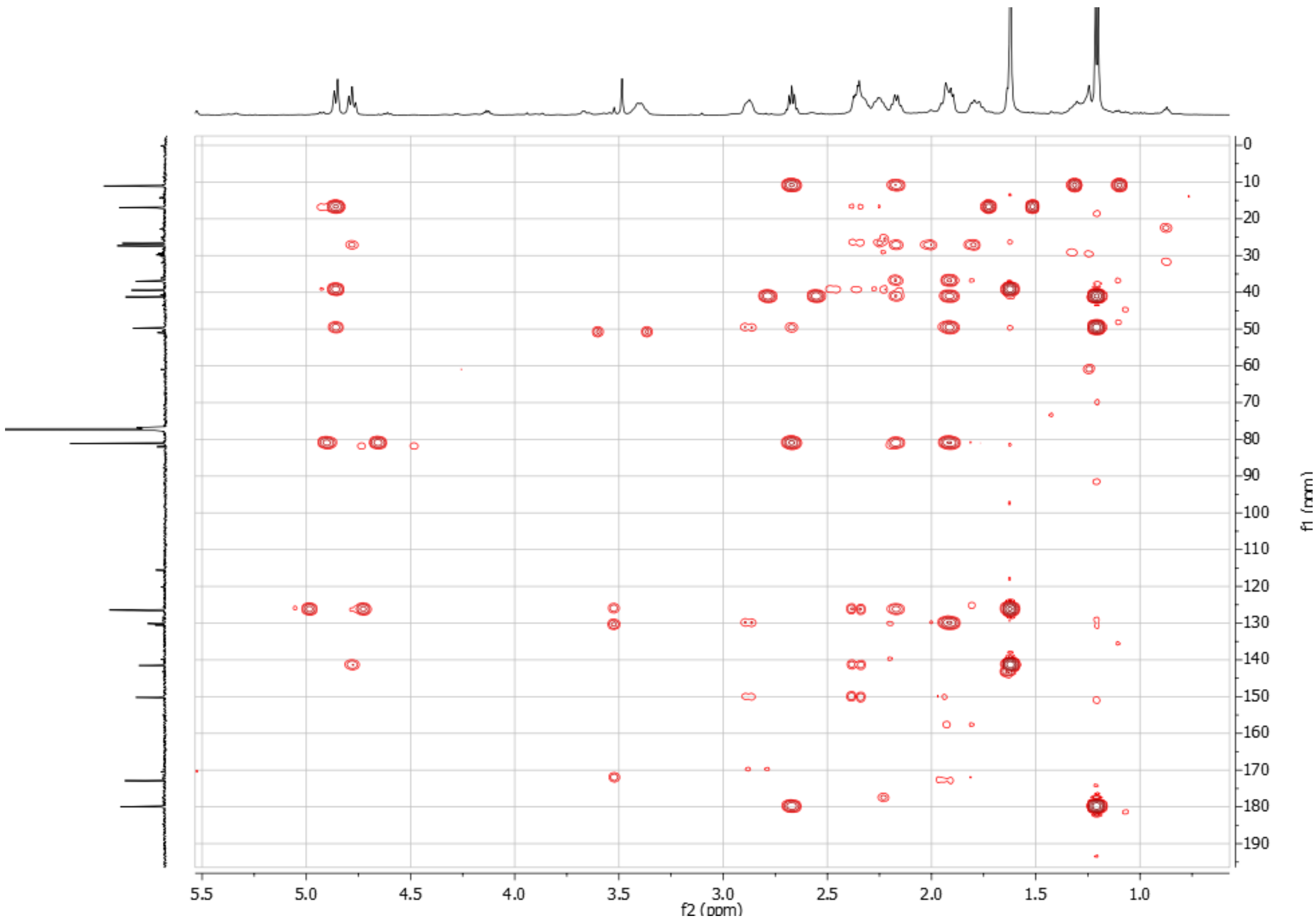

Fig. S5. ^{1}H–^{1}H COSY spectrum of compound **1** in $CDCl_3$

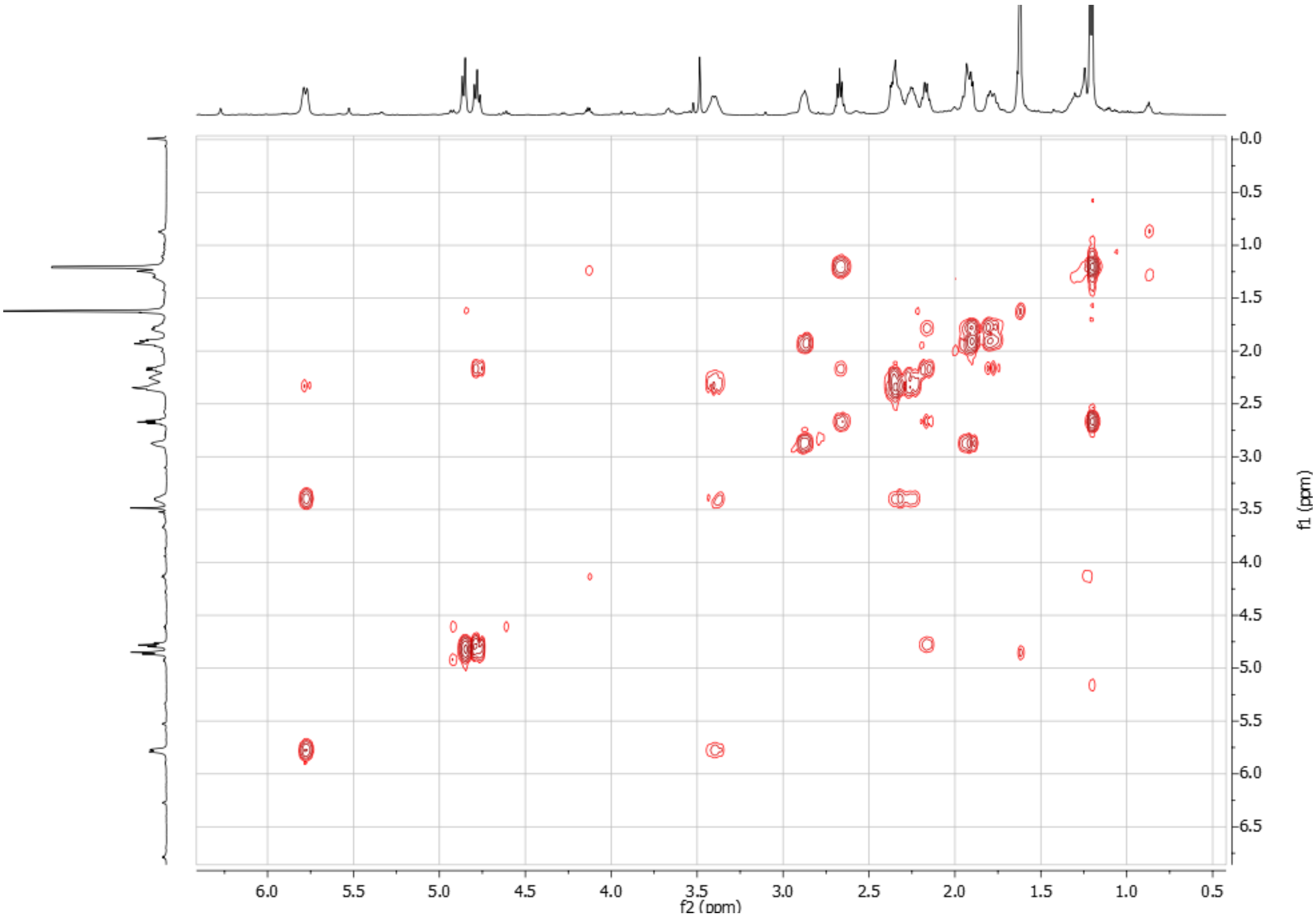

Fig. S6. NOESY spectrum of compound **1** in $CDCl_3$

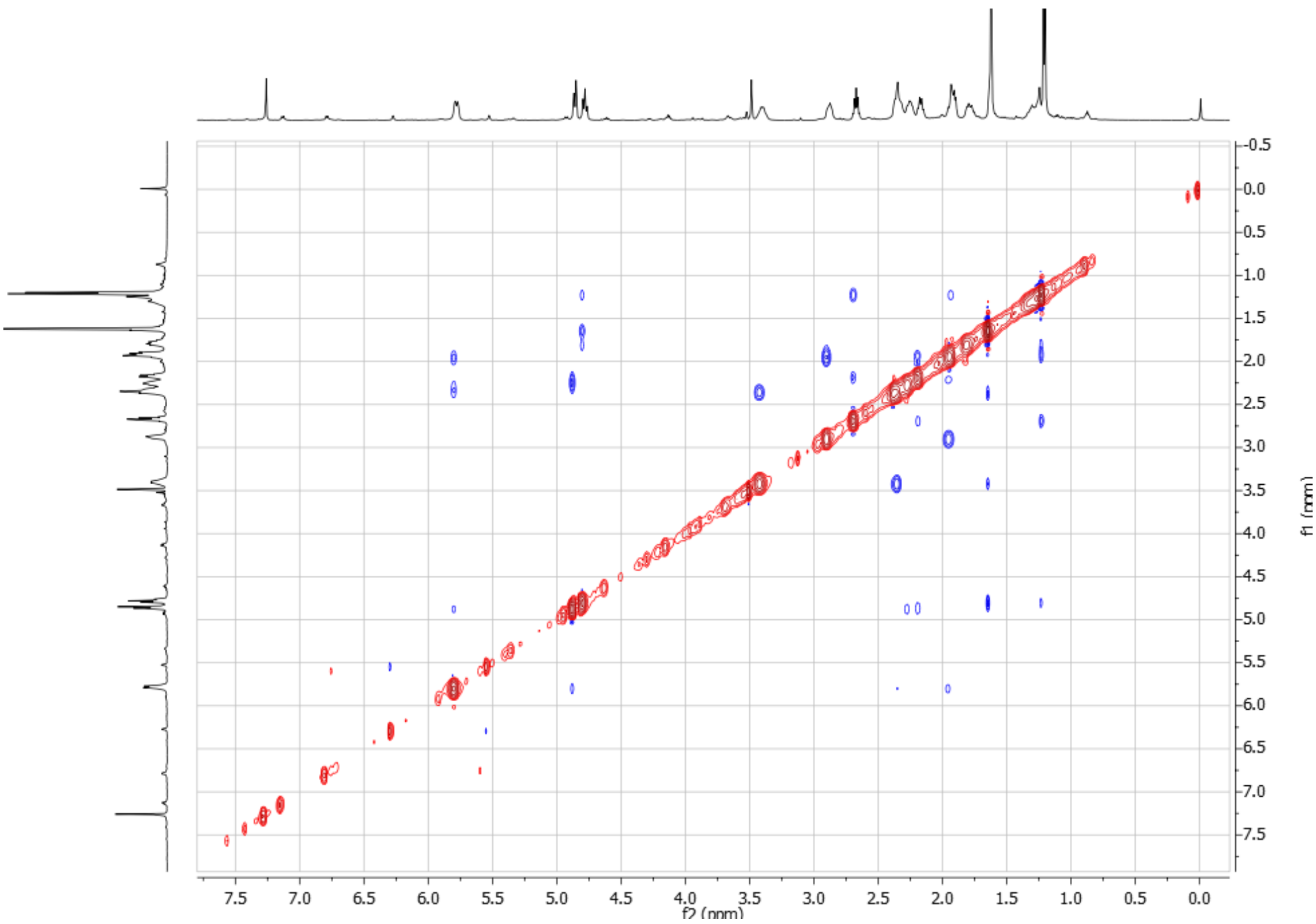

Fig. S7. (±)-ESIMS spectra of compound **1**

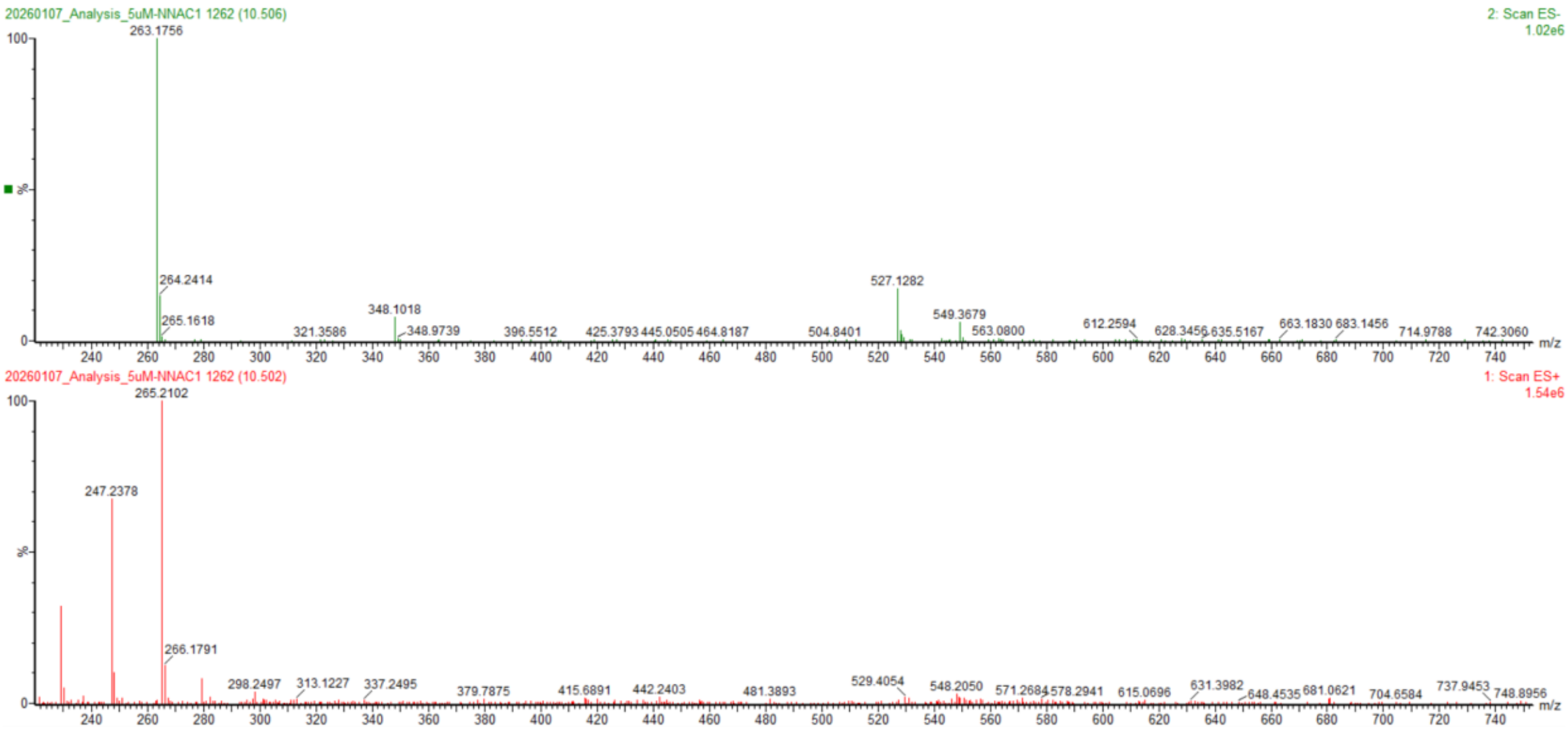

Fig. S8. (+)-HRESIMS spectrum of compound **1**

Qualitative Analysis Report

**Data Filename**	N_ESIH202504464.d	**Sample Name**	D4-T10D
**Sample ID**		**Position**	P1-A7
**Instrument Name**	Agilent 6520 Q-TOF	**Acq Method**	20160324_MS_ESIH_NEG_1min.m
**Acquired Time**	9/3/2025 9:18:20 AM	**DA Method**	ESI-HR-20231114.m
**Comment**	ESIH by huangqiongping		

User Spectra

Fragmentor Voltage	Collision Energy	Ionization Mode
170	0	ESI

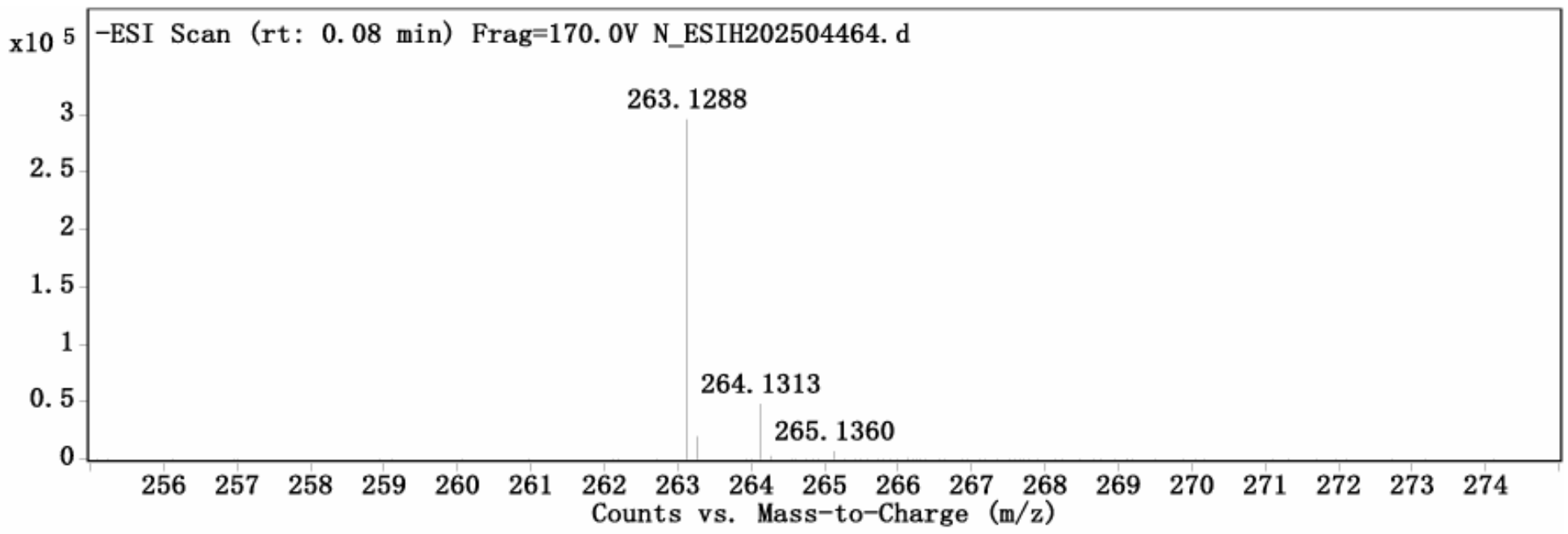

Formula Calculator Results

m/z	Calc m/z	Diff (mDa)	Diff (ppm)	Ion Formula	Ion
263.1288	263.1289	0.09	0.33	C15 H19 O4	(M-H)-

Fig. S9. IR spectrum of compound **1**

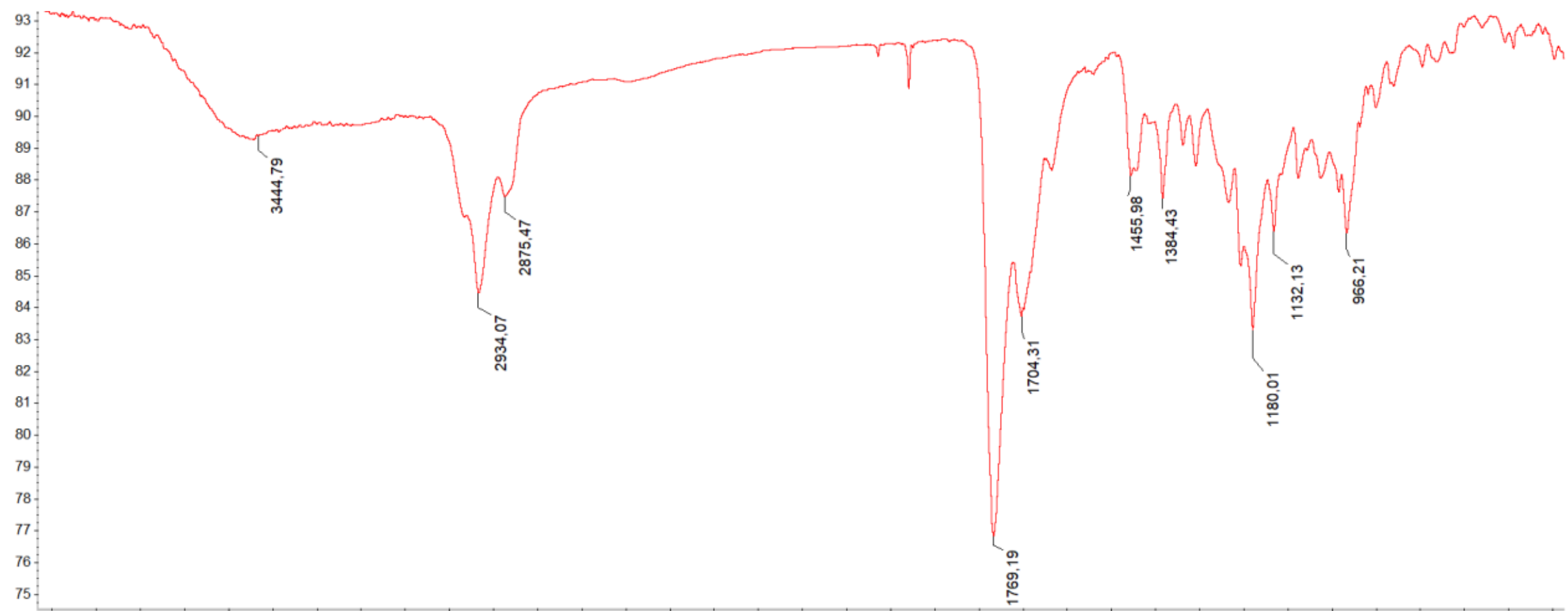

Fig. S10. ^{1}H NMR spectrum of compound **2** in $CDCl_3$

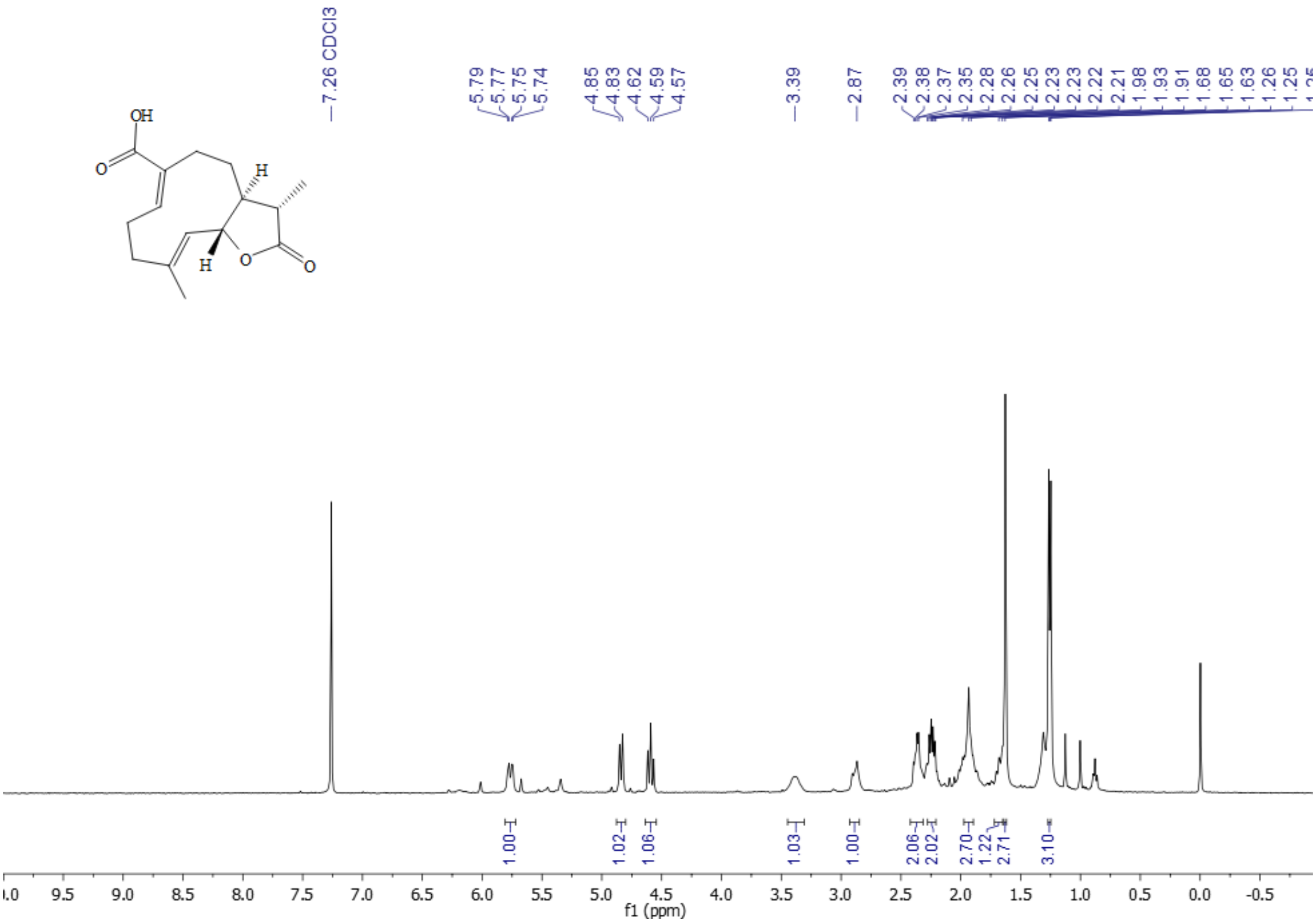

Fig. S11. ^{1}H NMR spectrum of compound **2** in methanol-d_4

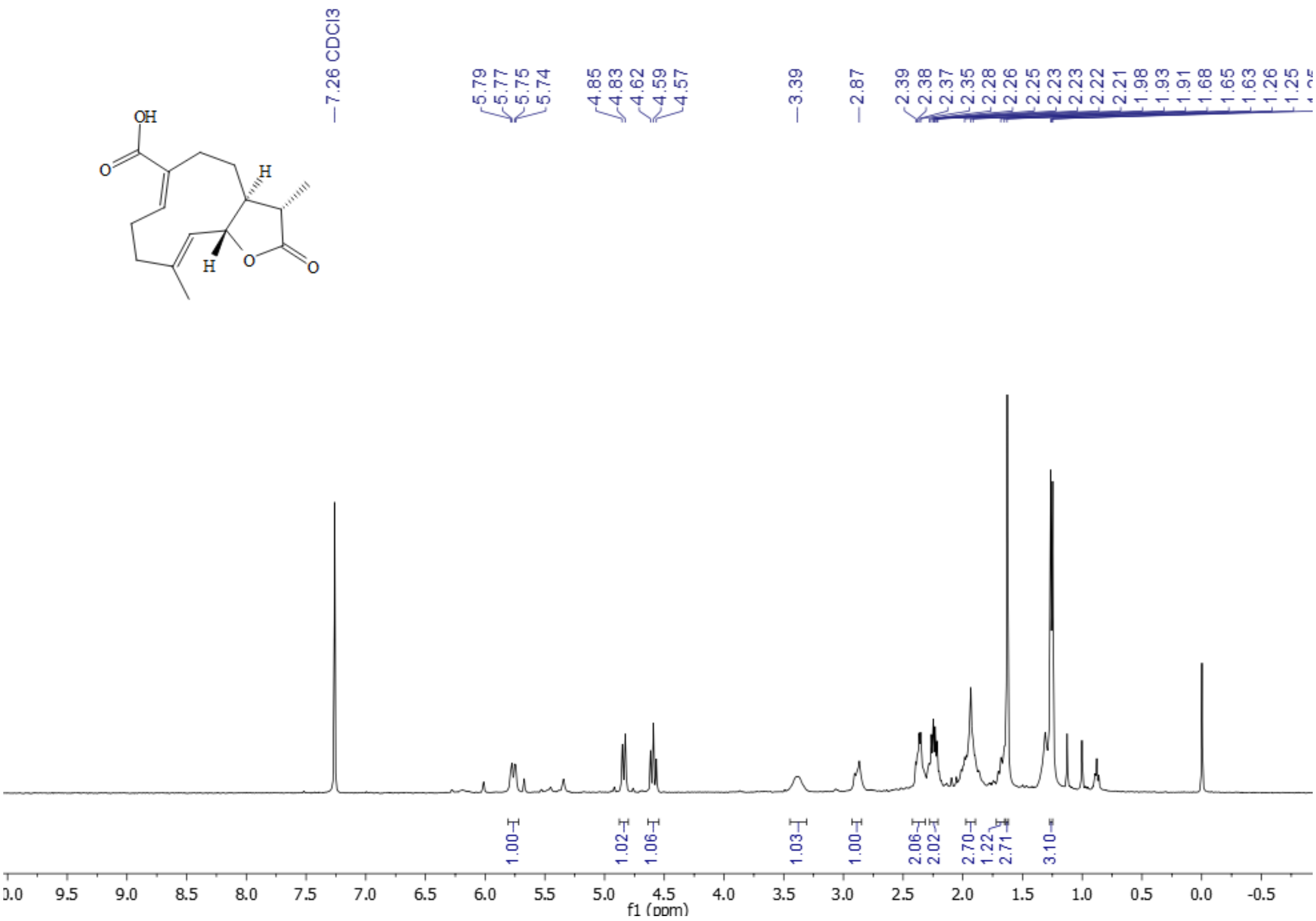

Fig. S12. ^{13}C NMR spectrum of compound **2** in methanol-d_4

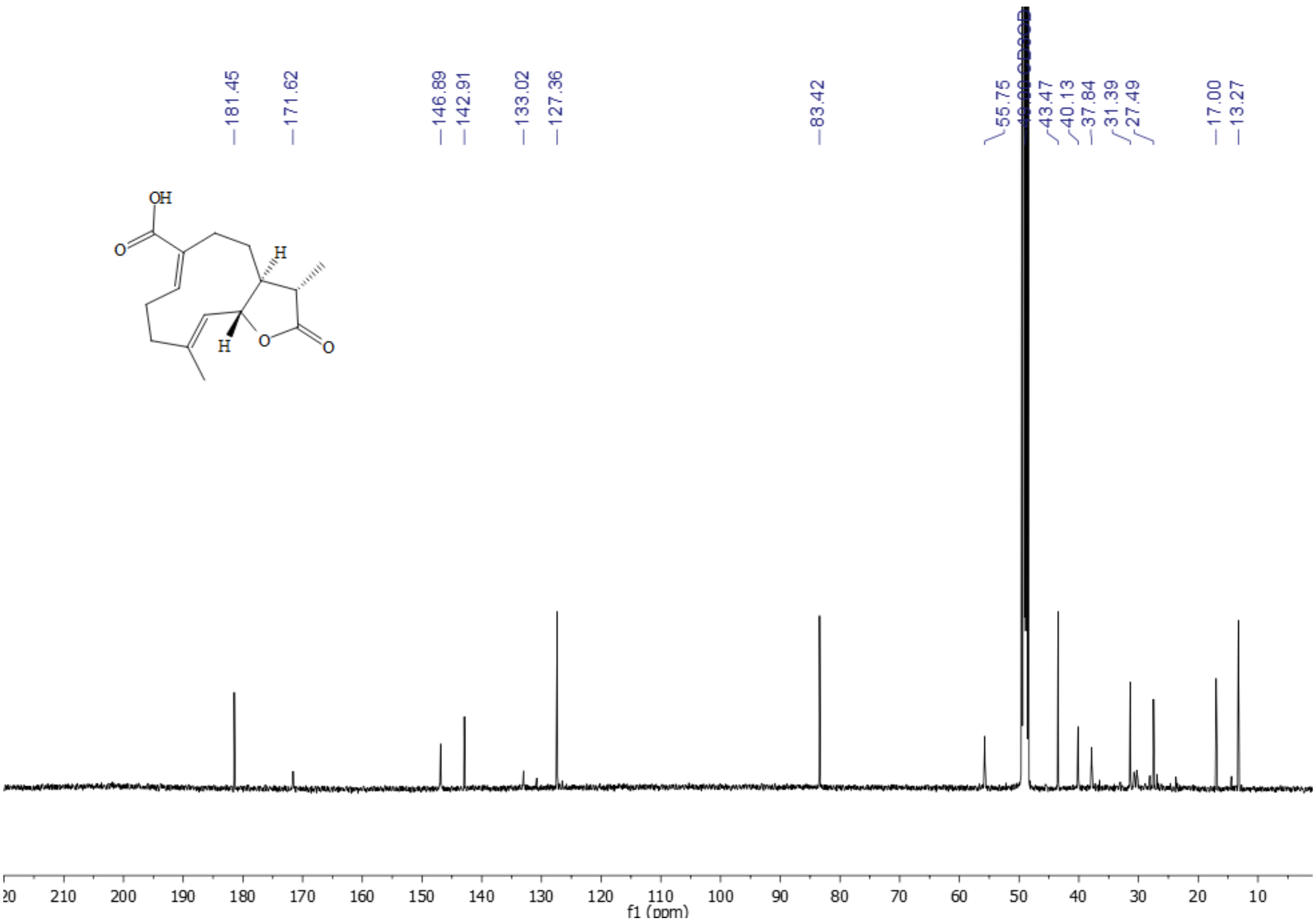

Fig. S13. (±)-ESIMS spectra of compound **2**

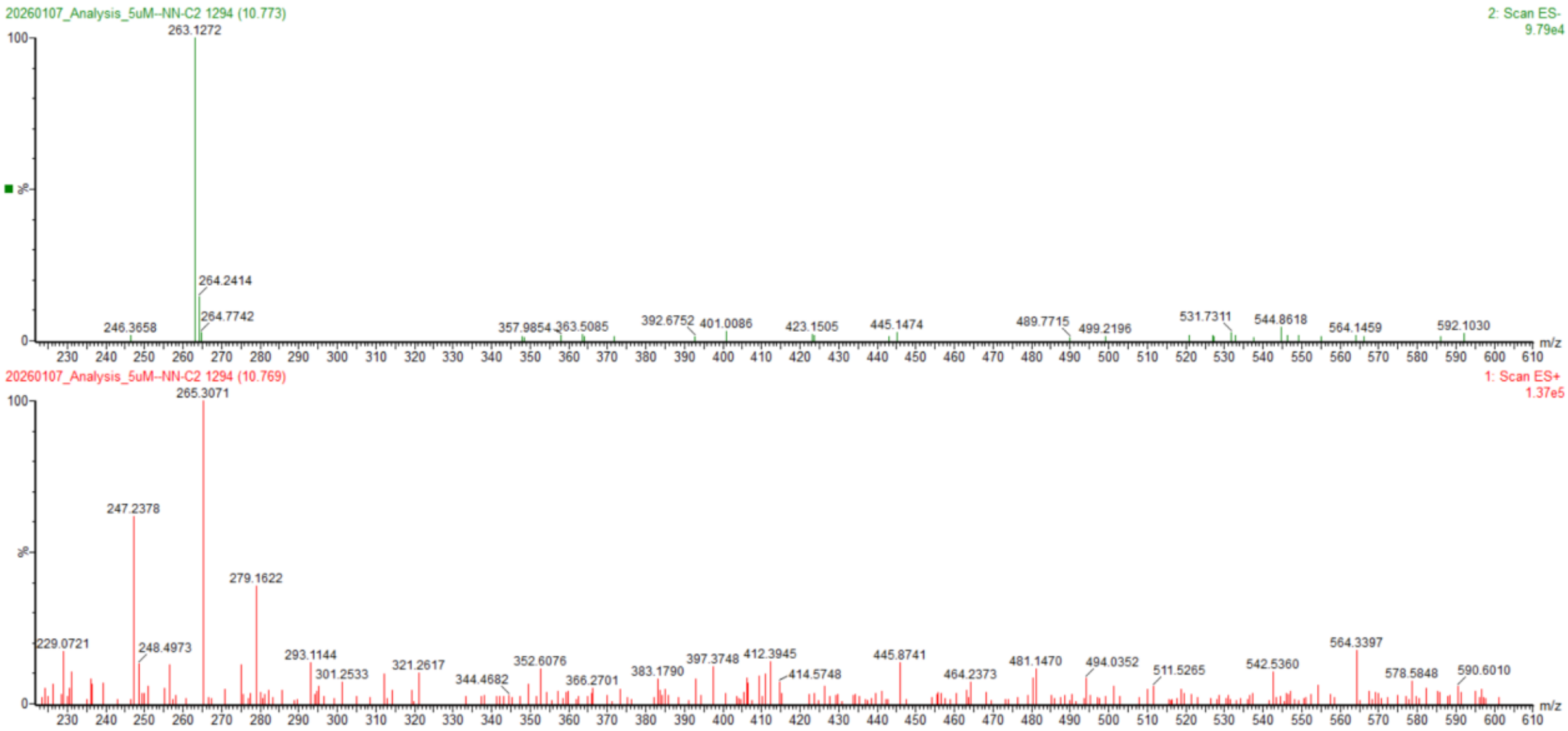

Fig. S14. ^{1}H NMR spectrum of compound **3** in $CDCl_3$

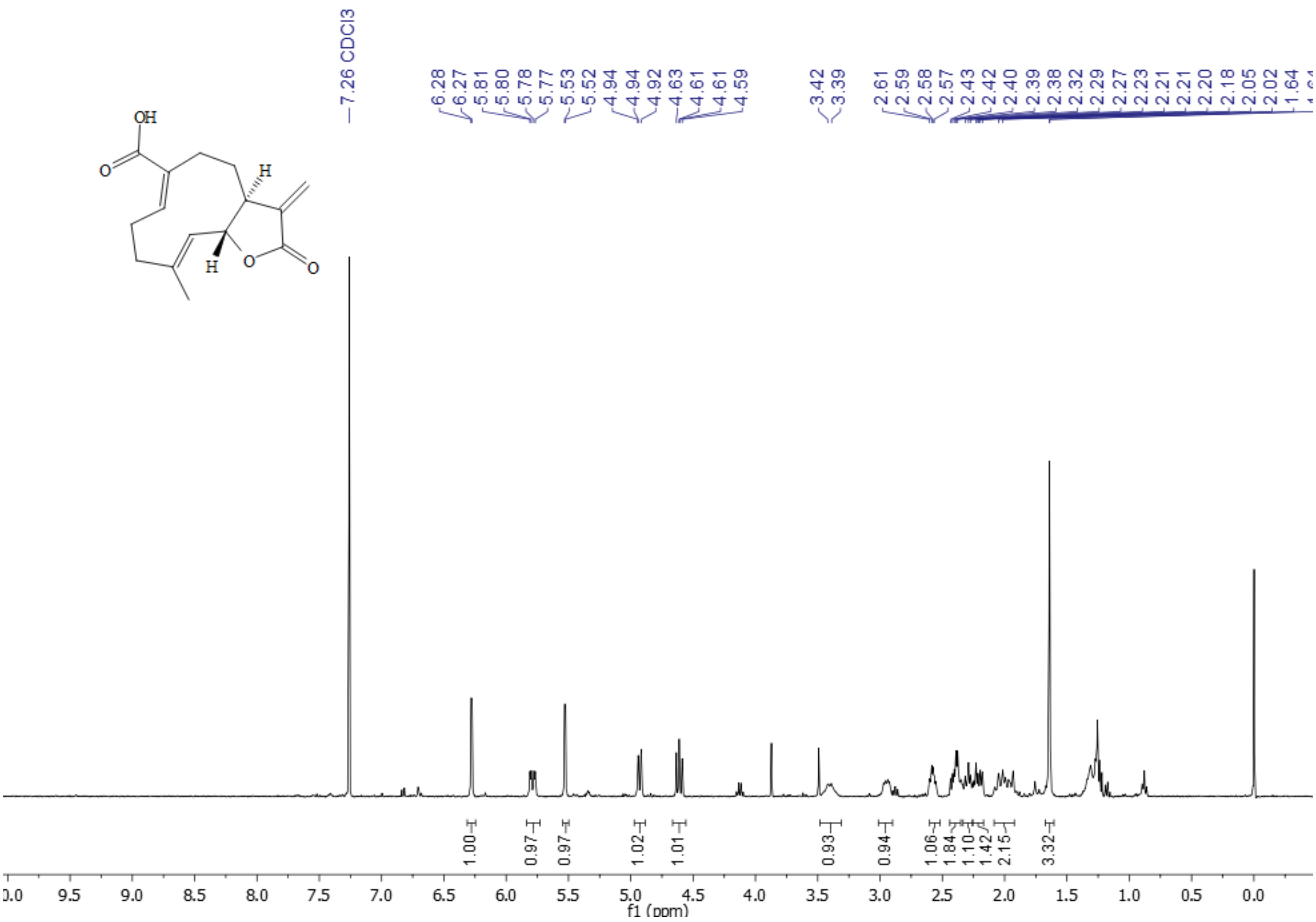

Fig. S15. (±)-ESIMS spectra of compound **3**

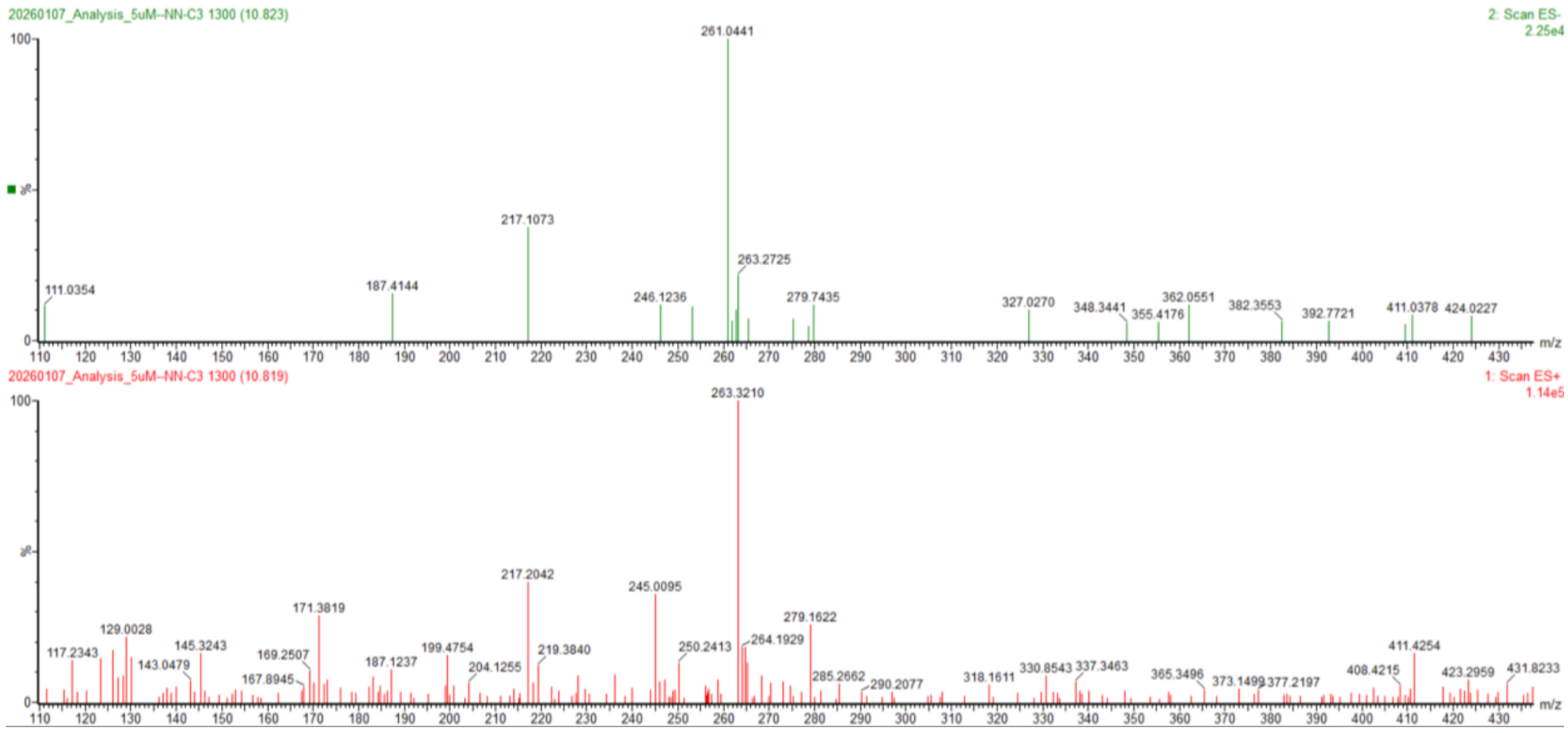

Fig. S16. ^{1}H NMR spectrum of compound **4** in methanol-d_4

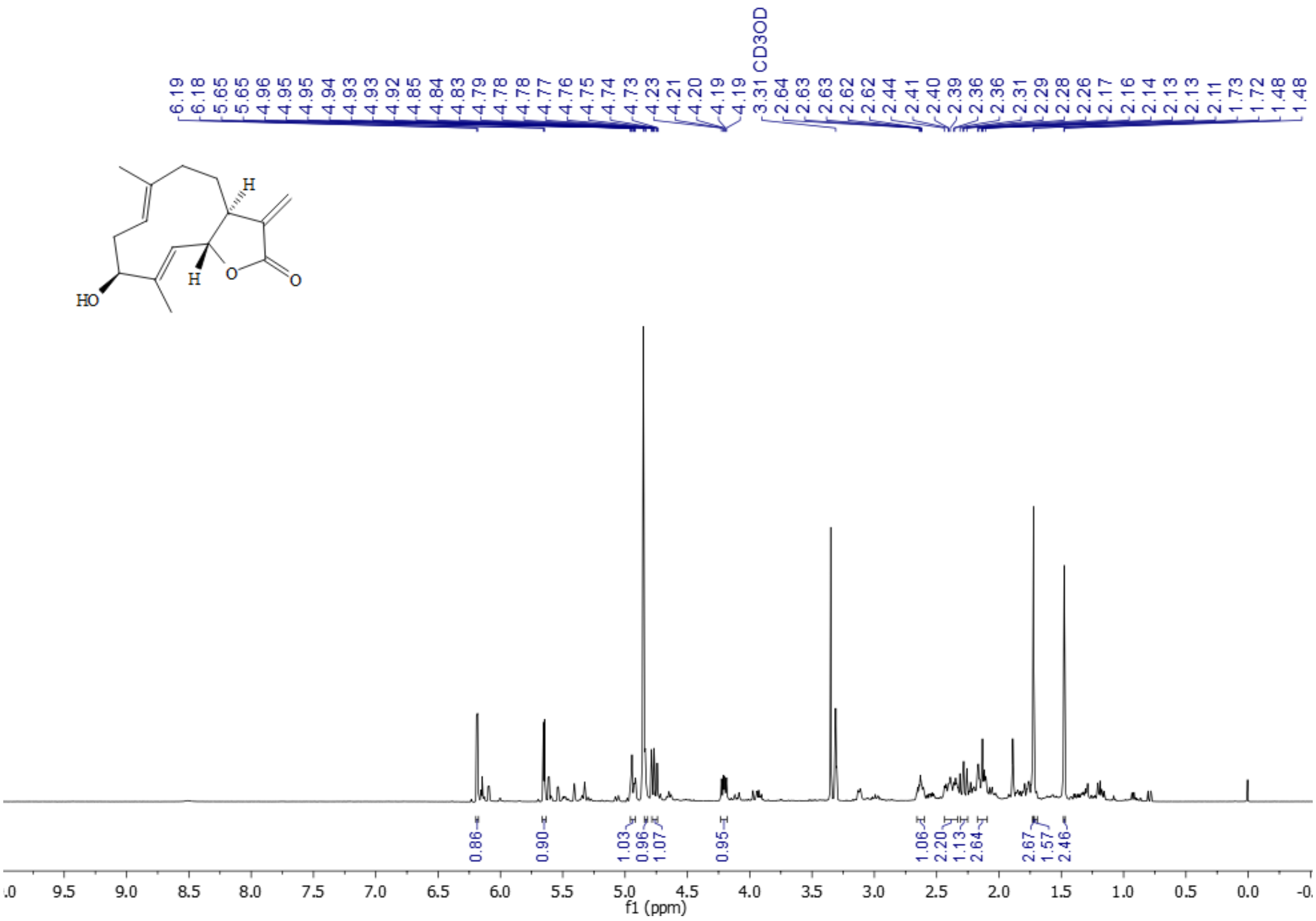

Fig. S17. ^{13}C NMR spectrum of compound **4** in methanol-d_4

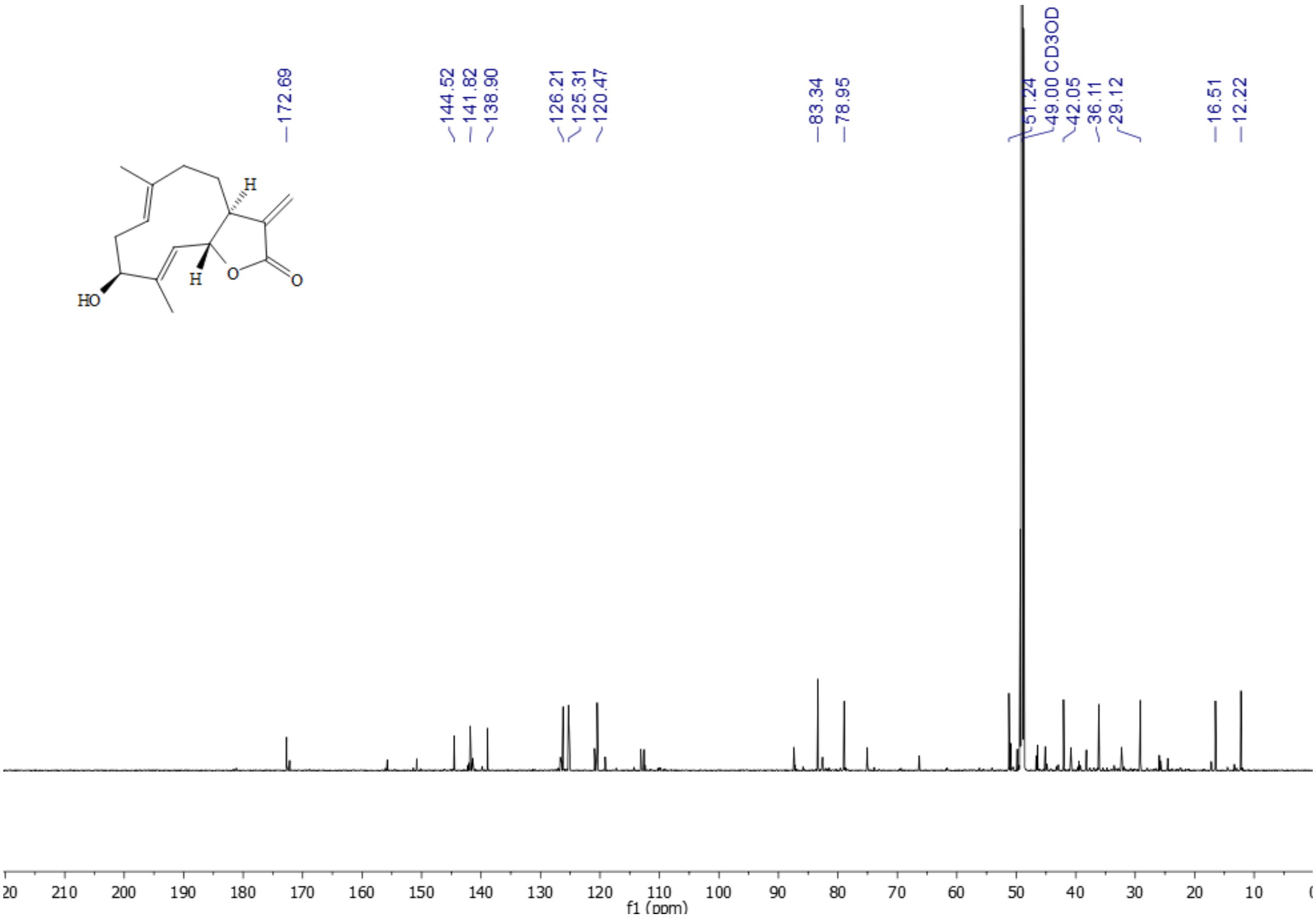

Fig. S18. (±)-ESIMS spectra of compound **4**

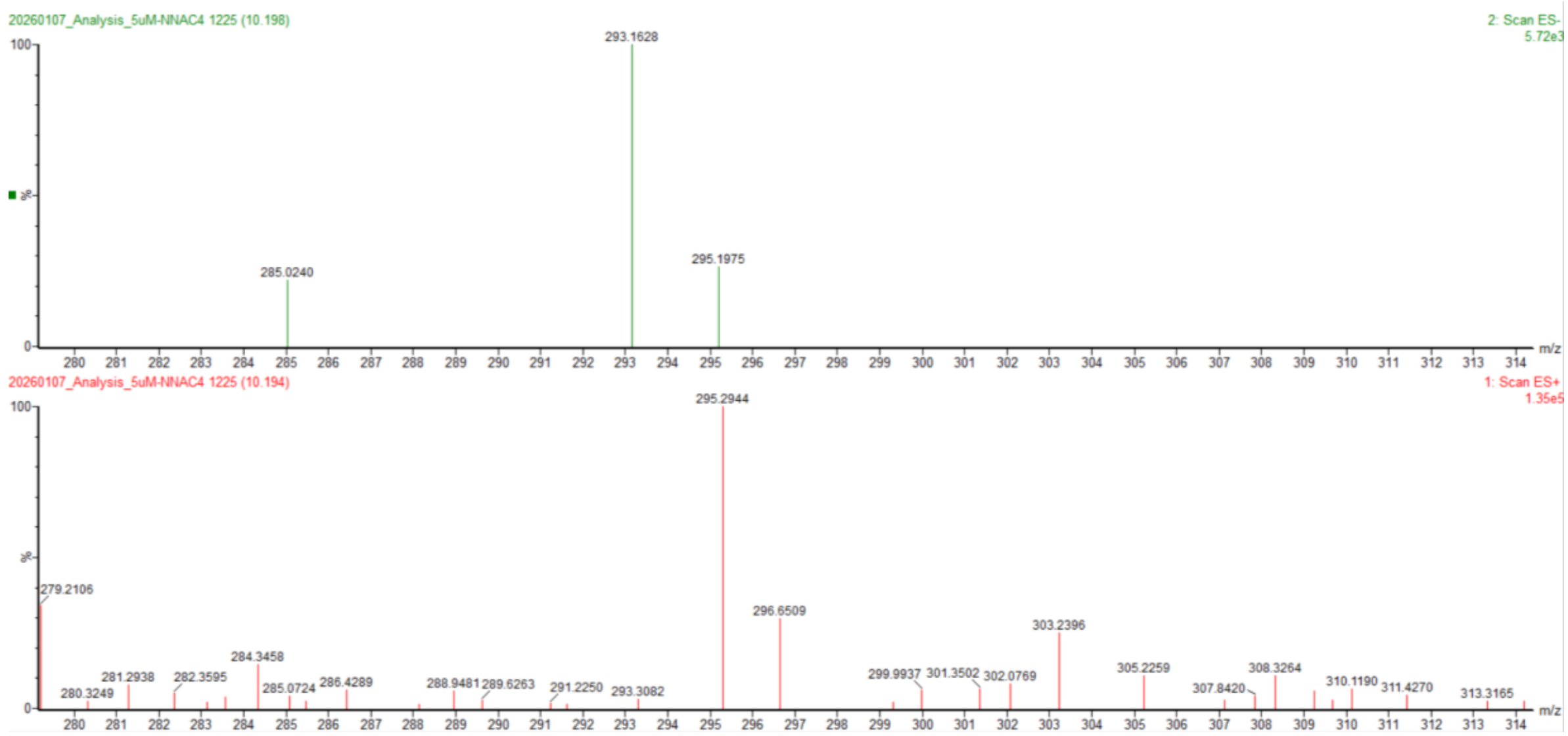

Fig. S19. ^{1}H NMR spectrum of compound **5** in DMSO-d_6

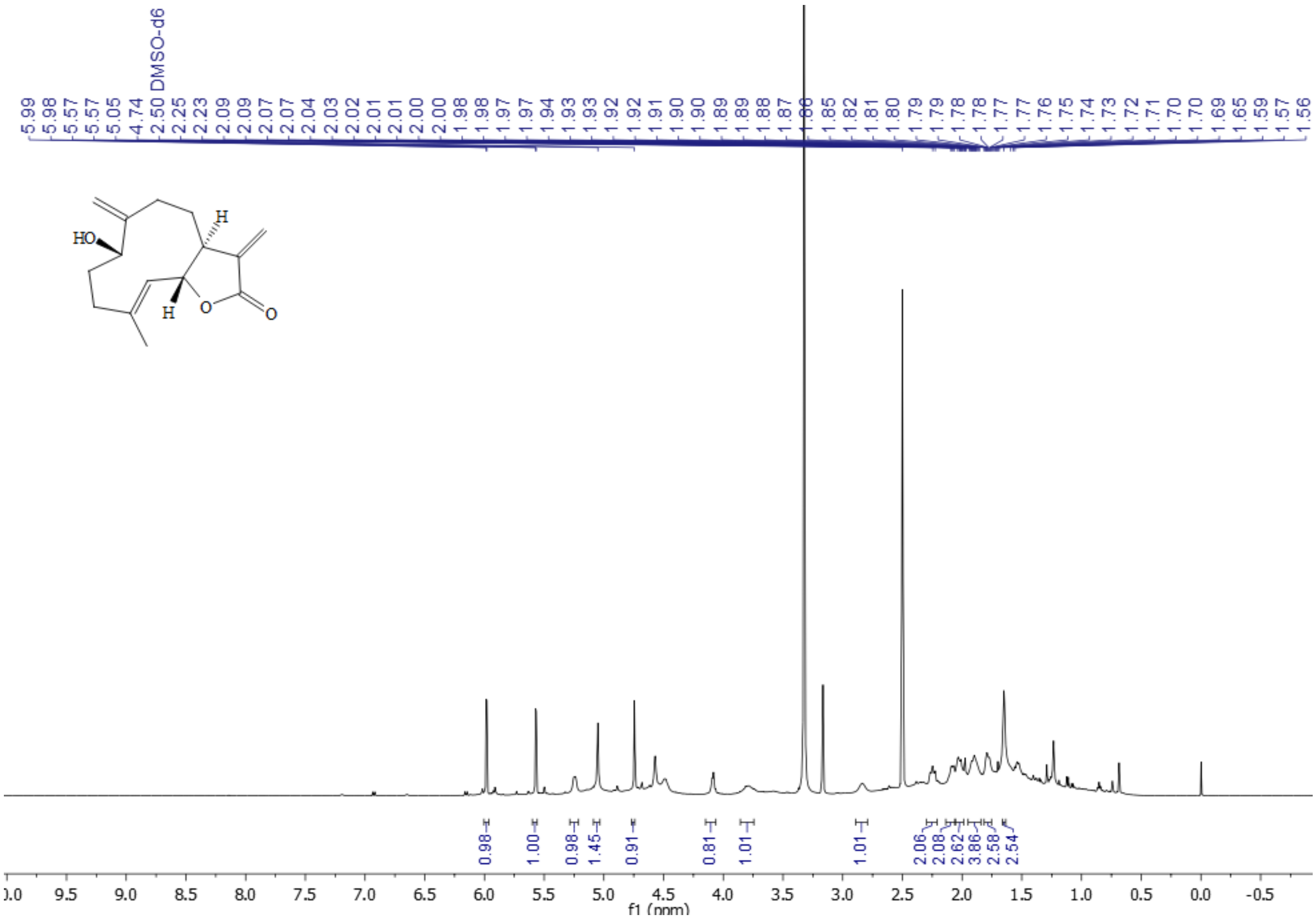

Fig. S20. ^{13}C NMR spectrum of compound **5** in DMSO-d_6

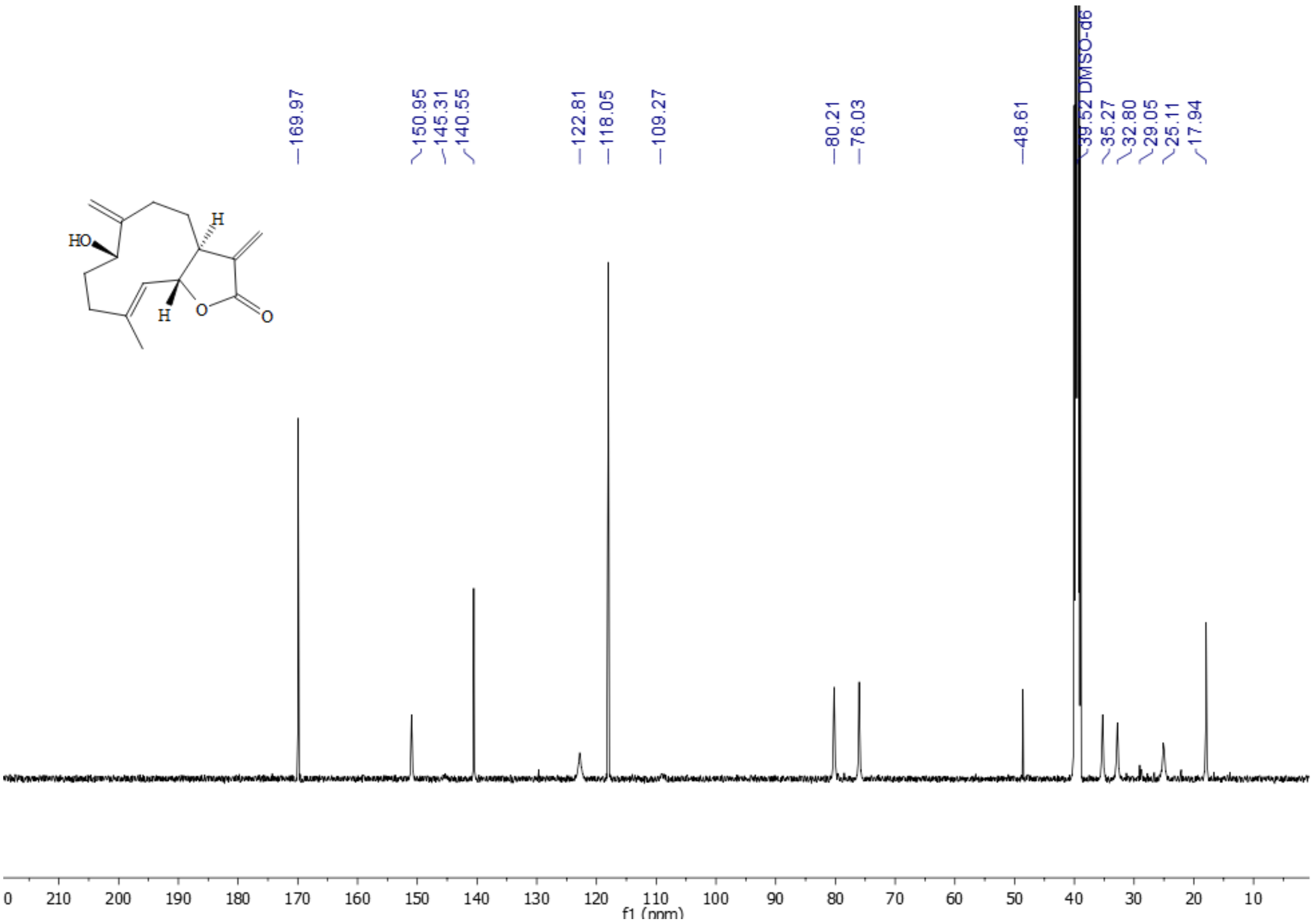

Fig. S21. (±)-ESIMS spectra of compound **5**

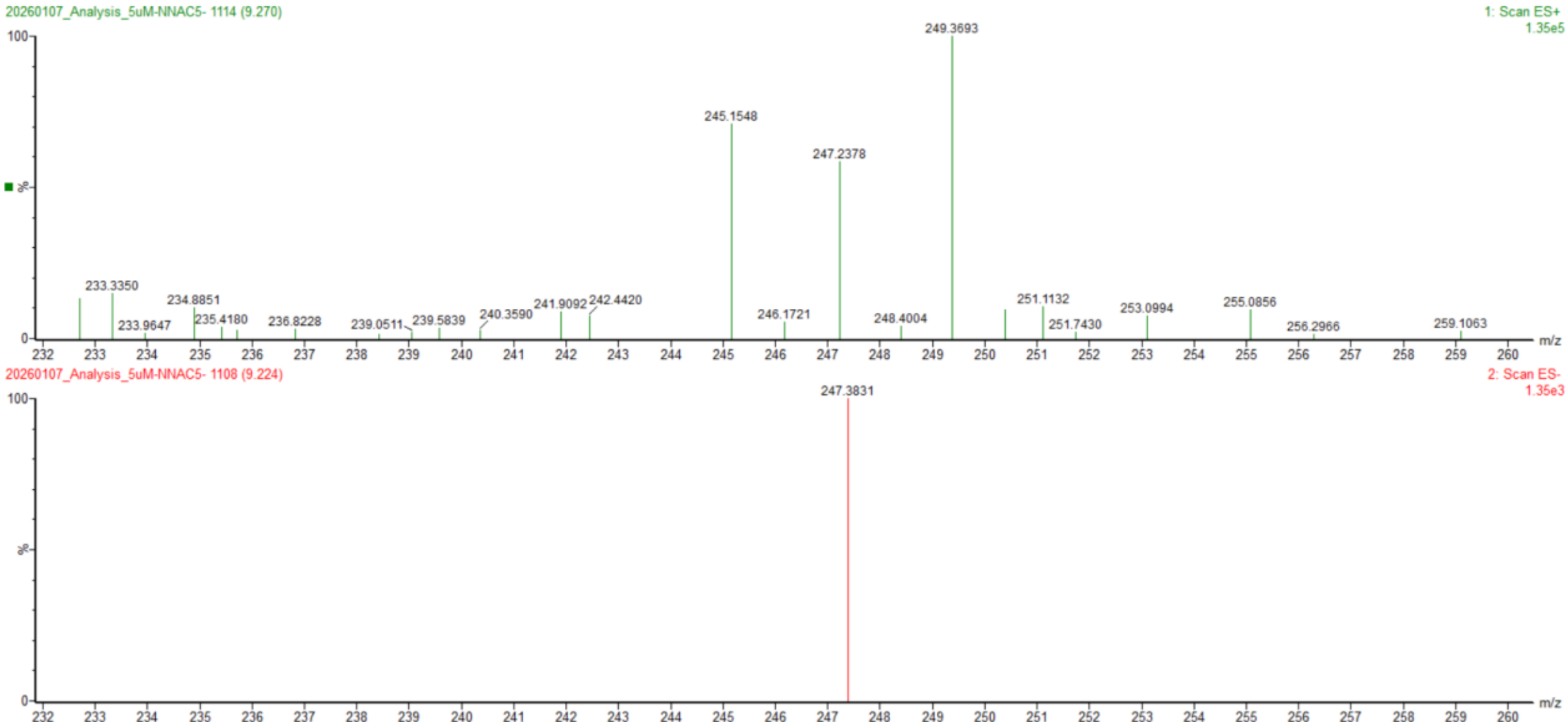

Fig. S22. ^{1}H NMR spectrum of compound **6** in methanol-d_4

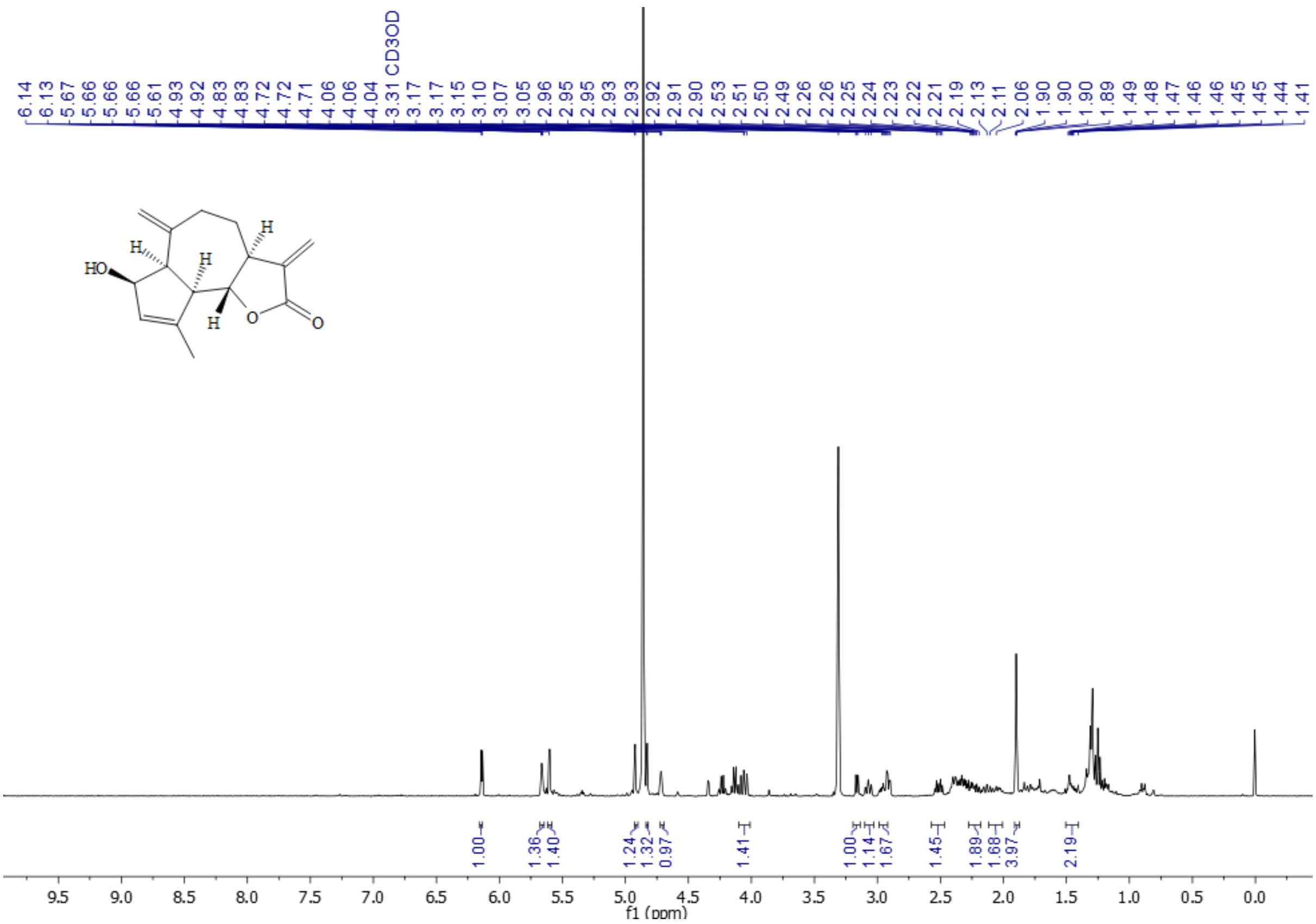

Fig. S23. ^{13}C NMR spectrum of compound **6** in methanol-d_4

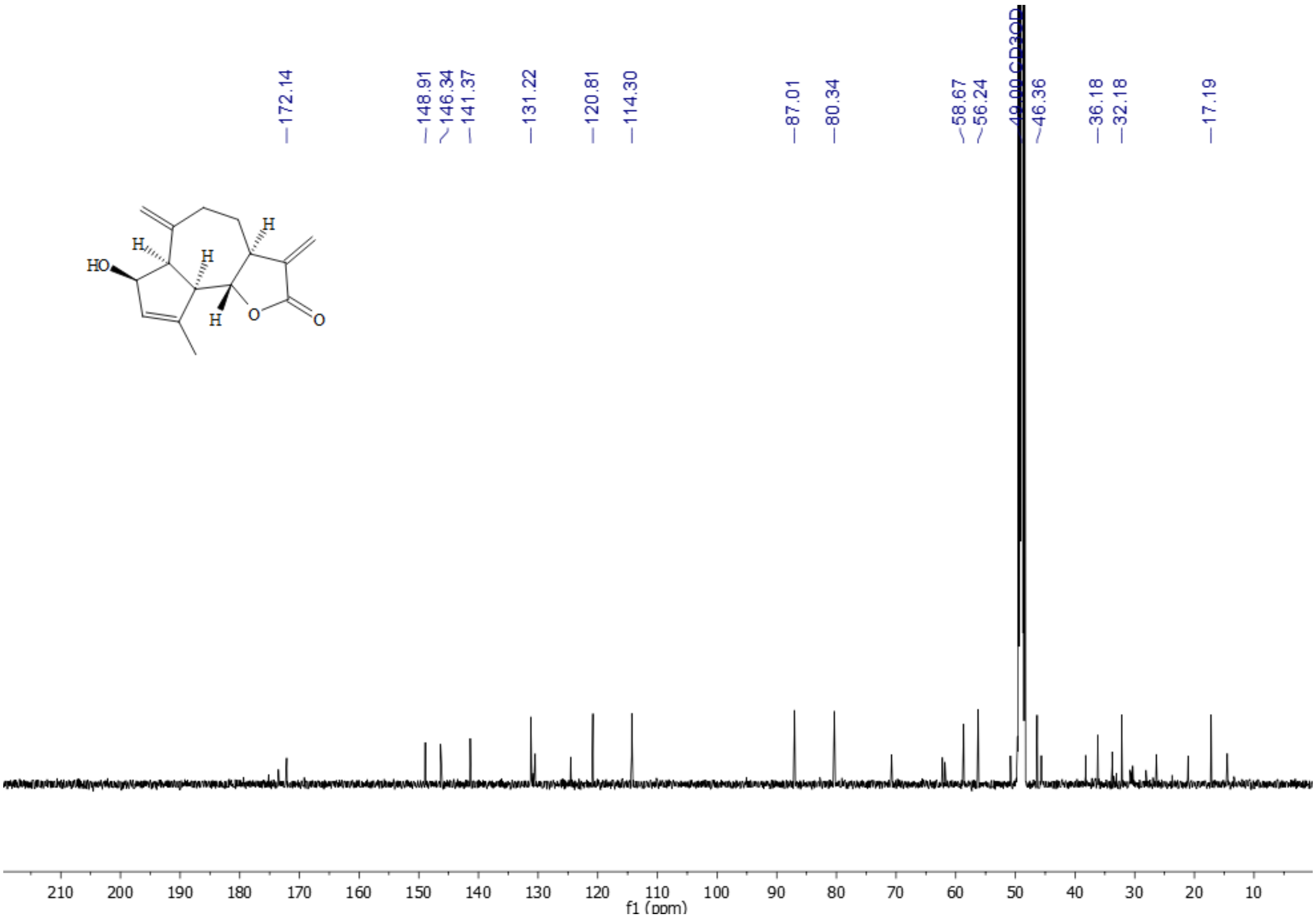

Fig. S24. (±)-ESIMS spectra of compound **6**

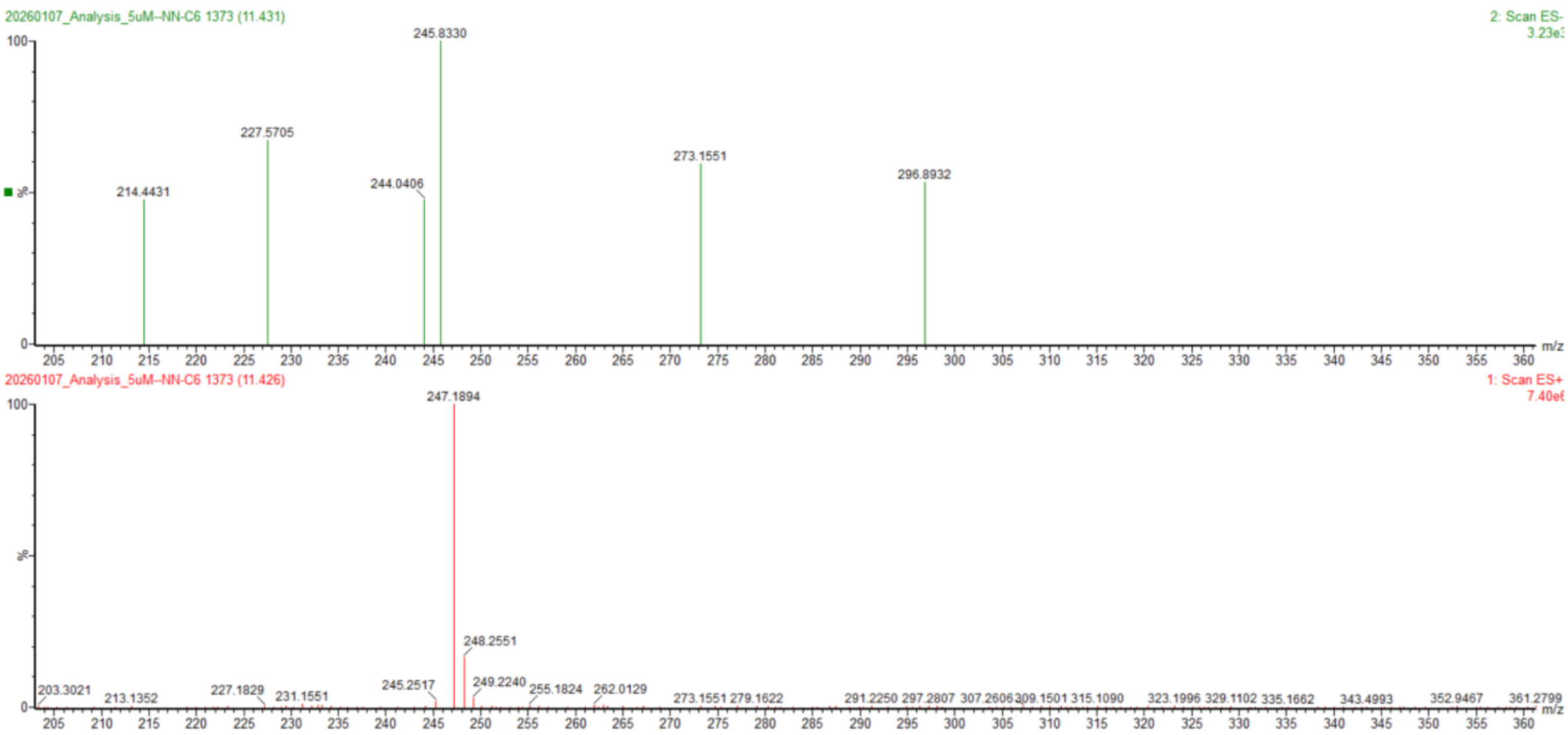

Fig. S25. ^{1}H NMR spectrum of compound **7** in $CDCl_3$

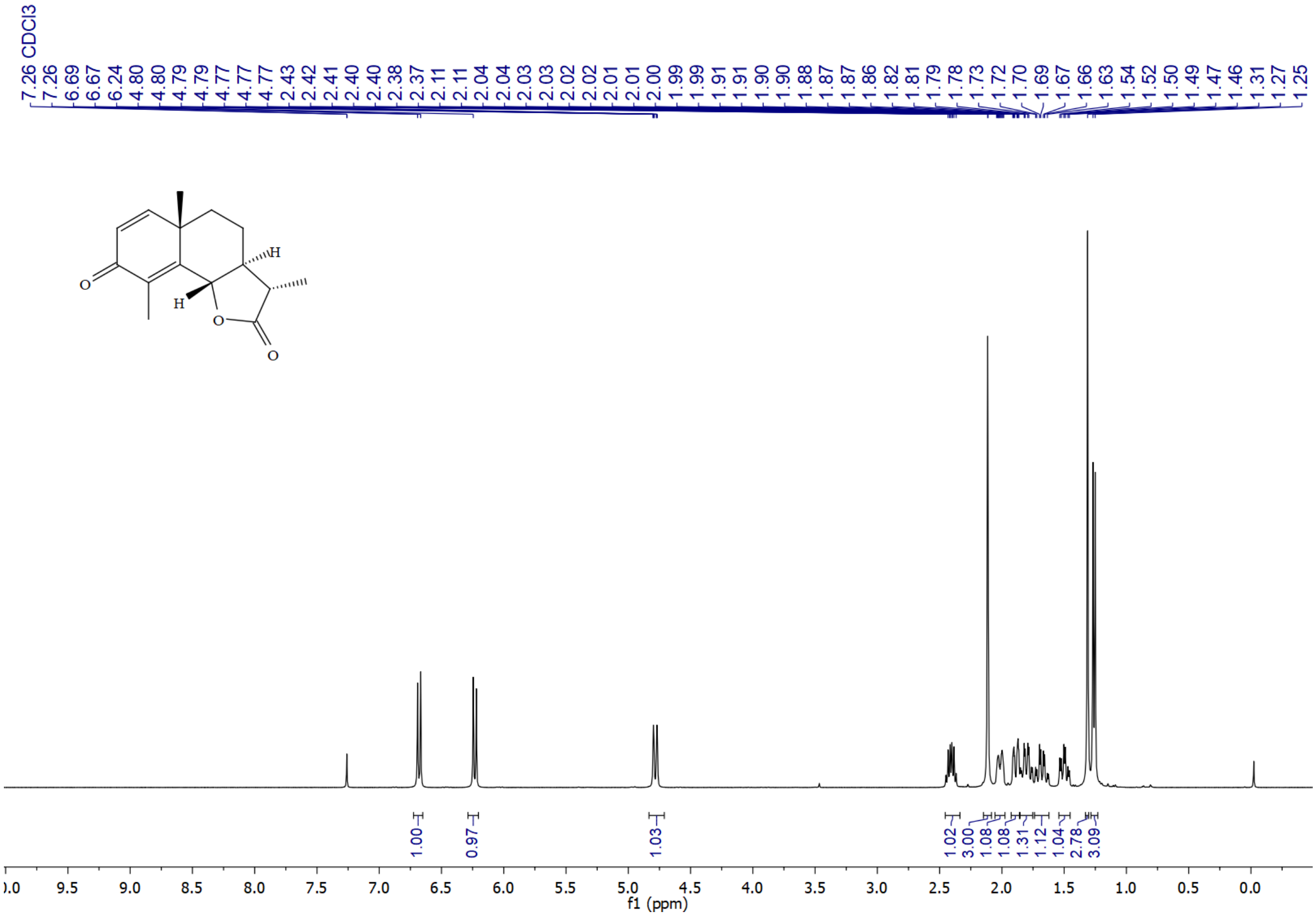

Fig. S26. ^{13}C NMR spectrum of compound **7** in $CDCl_3$

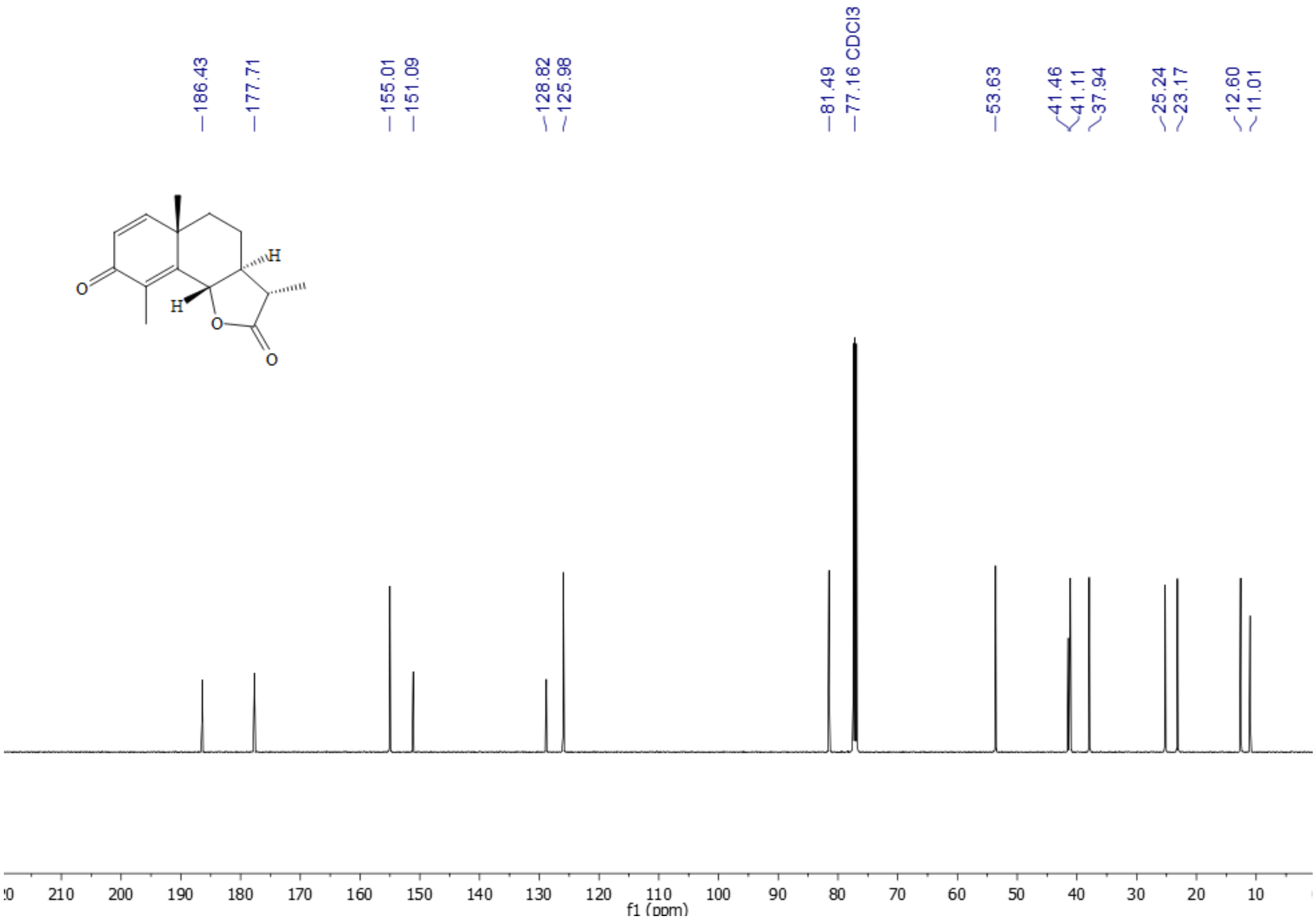

Fig. S27. (±)-ESIMS spectra of compound **7**

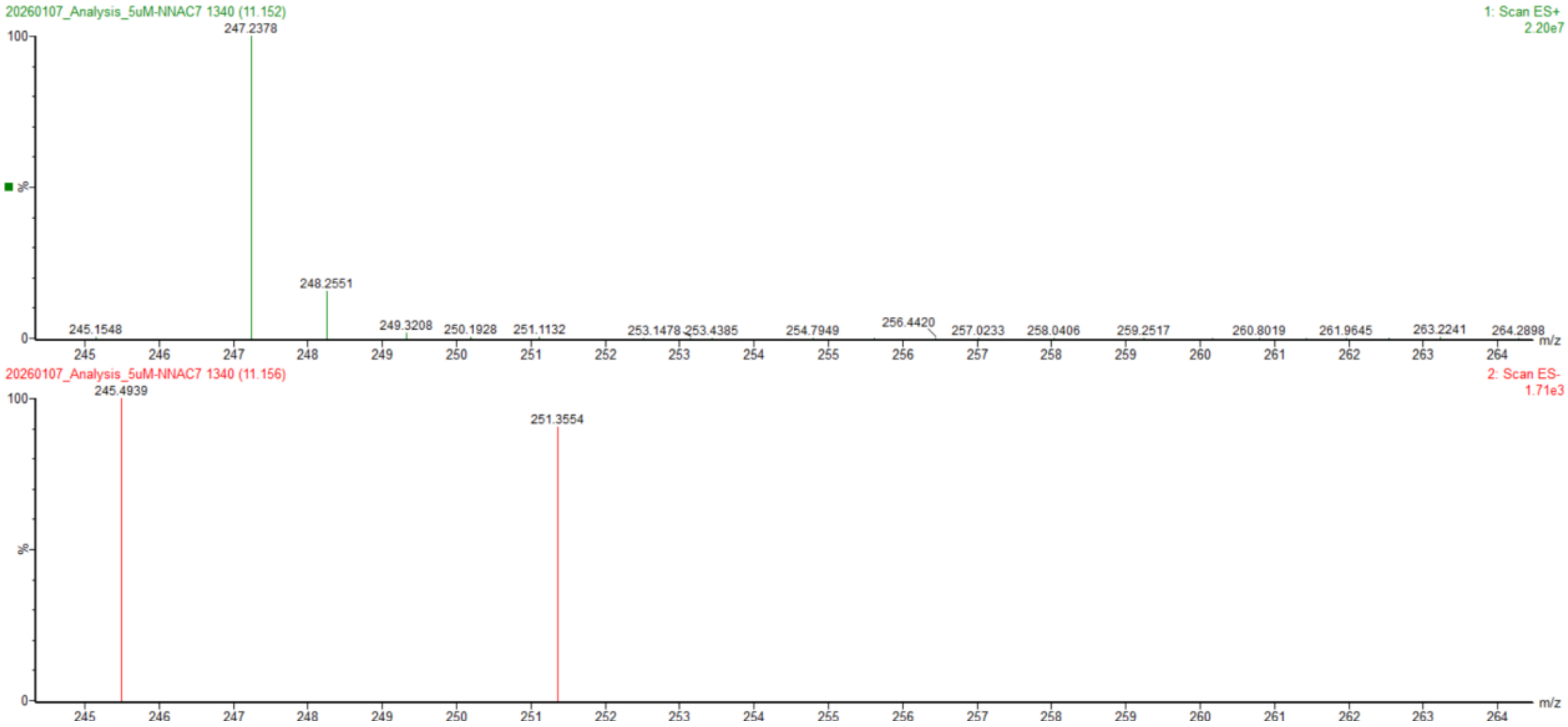

Fig. S28. ^{1}H NMR spectrum of compound **8** in $CDCl_3$

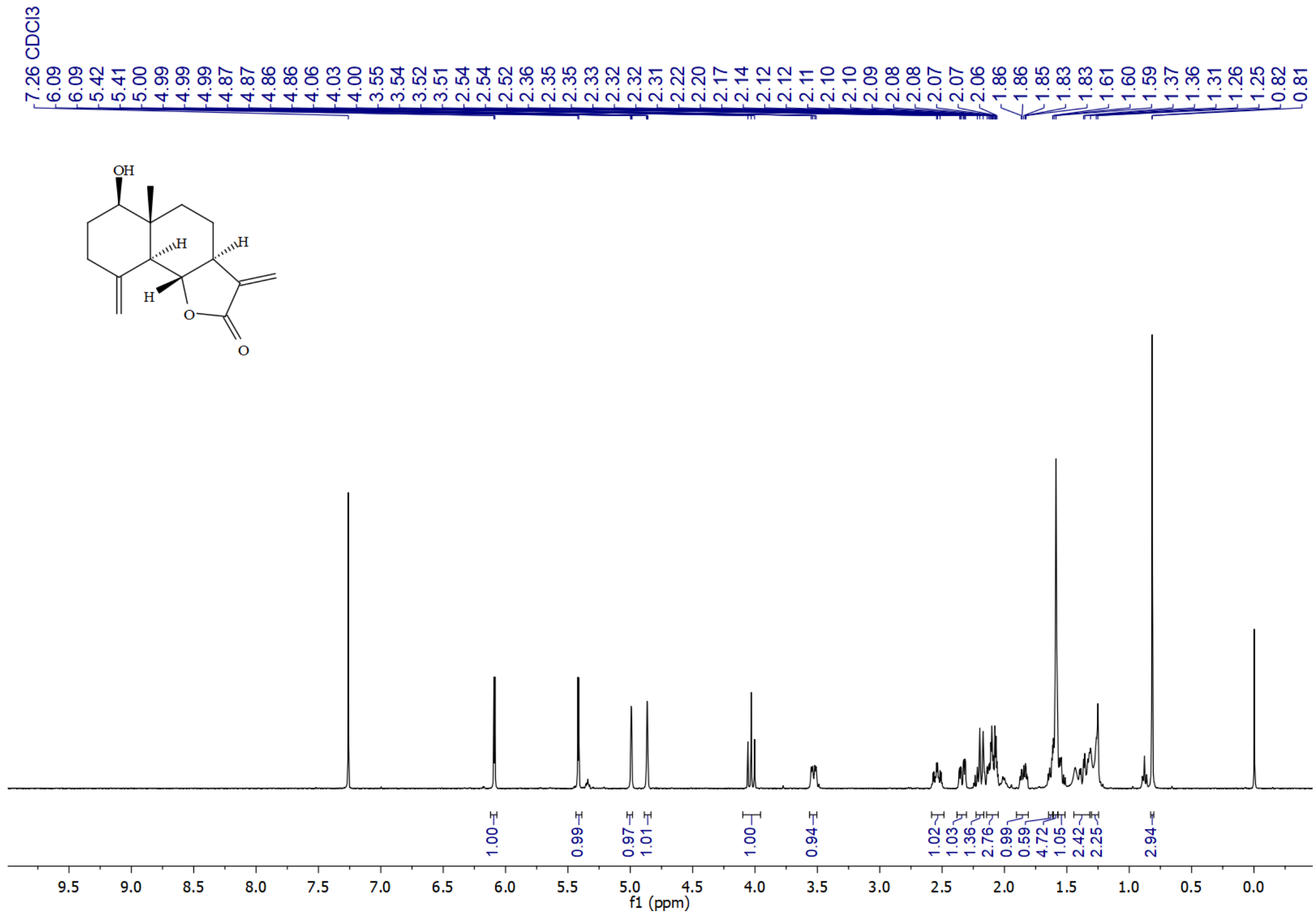

Fig. S29. ^{13}C NMR spectrum of compound **8** in $CDCl_3$

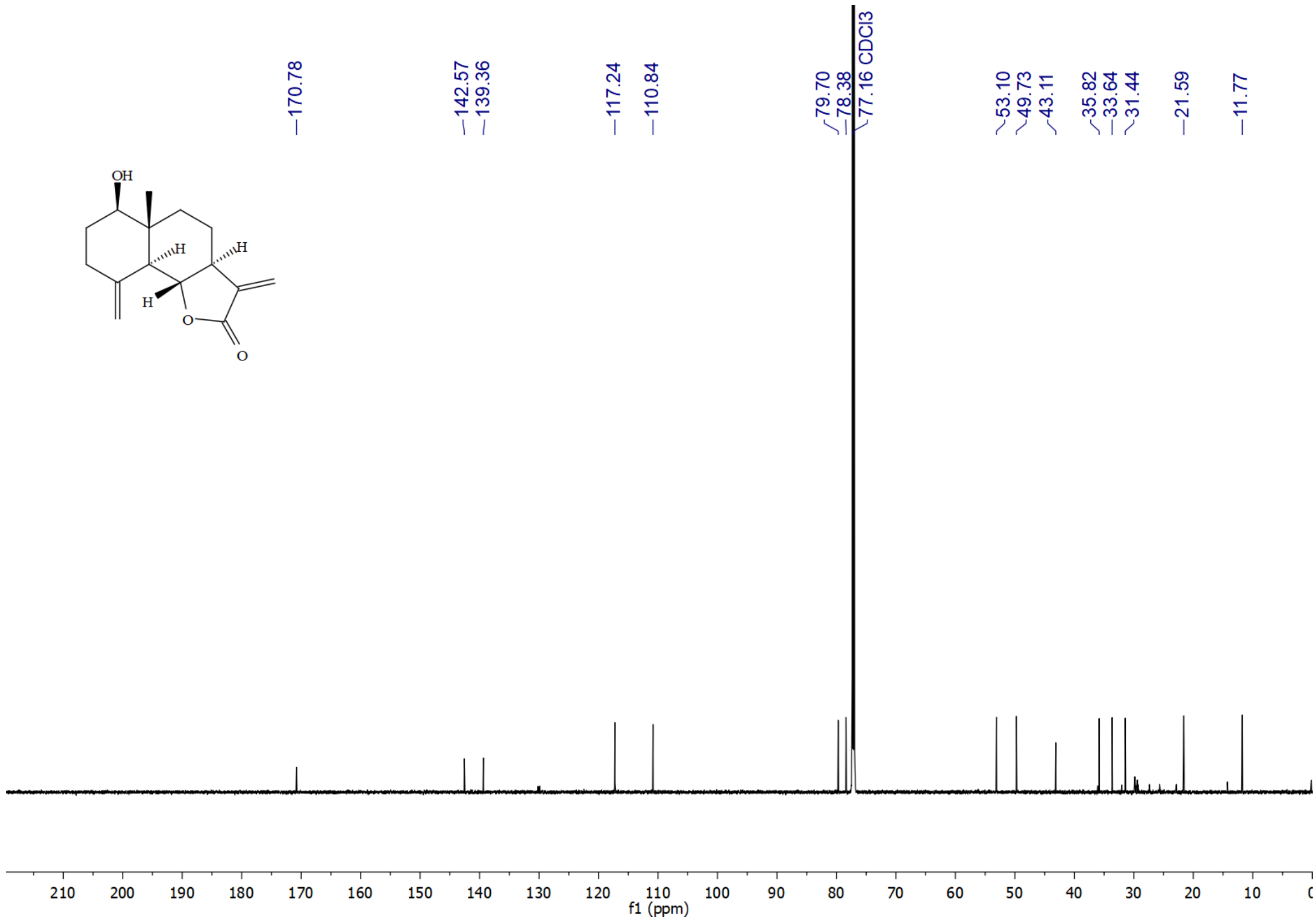

Fig. S30. (±)-ESIMS spectra of compound **8**

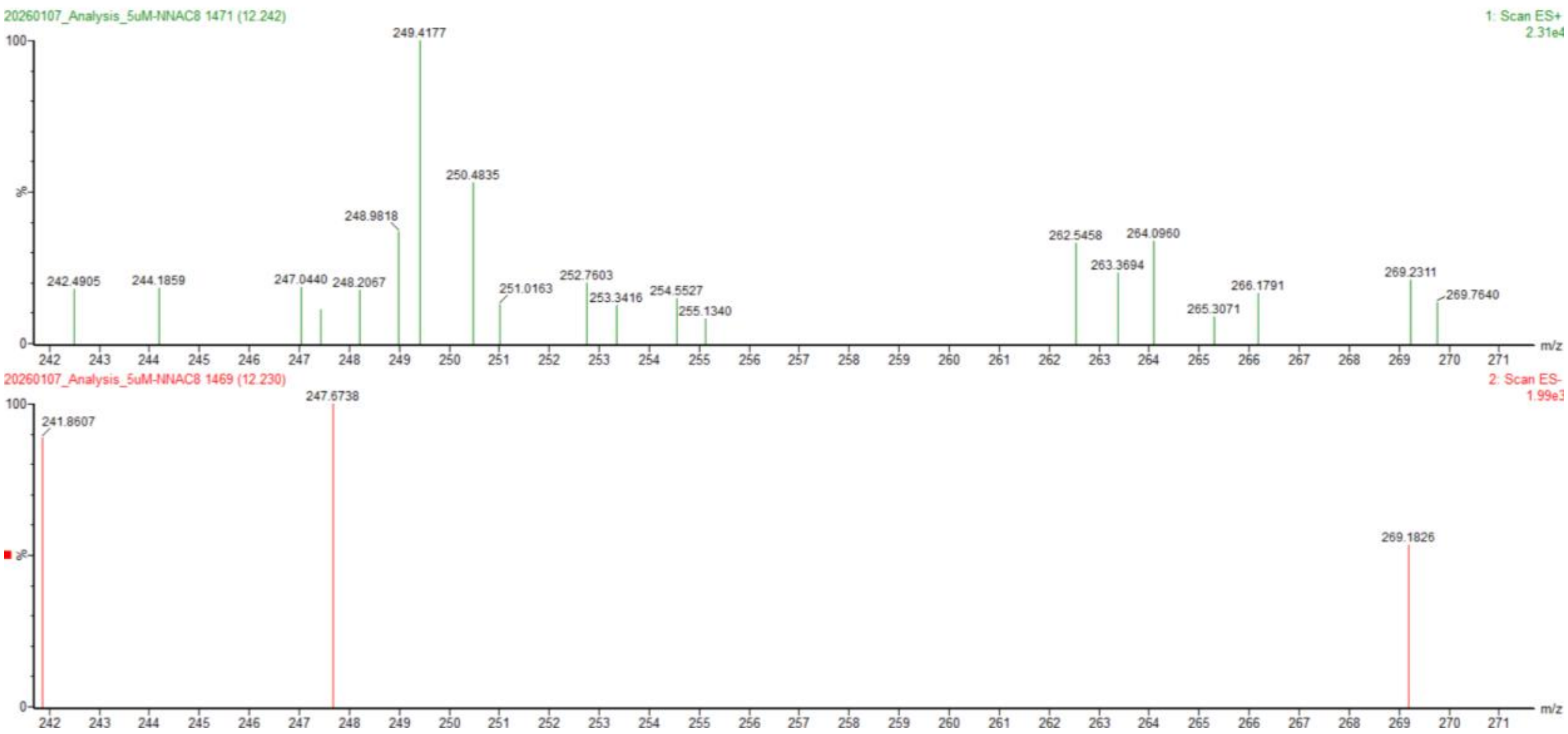

Fig. S31. ^{1}H NMR spectrum of compound **9** in methanol-d_4

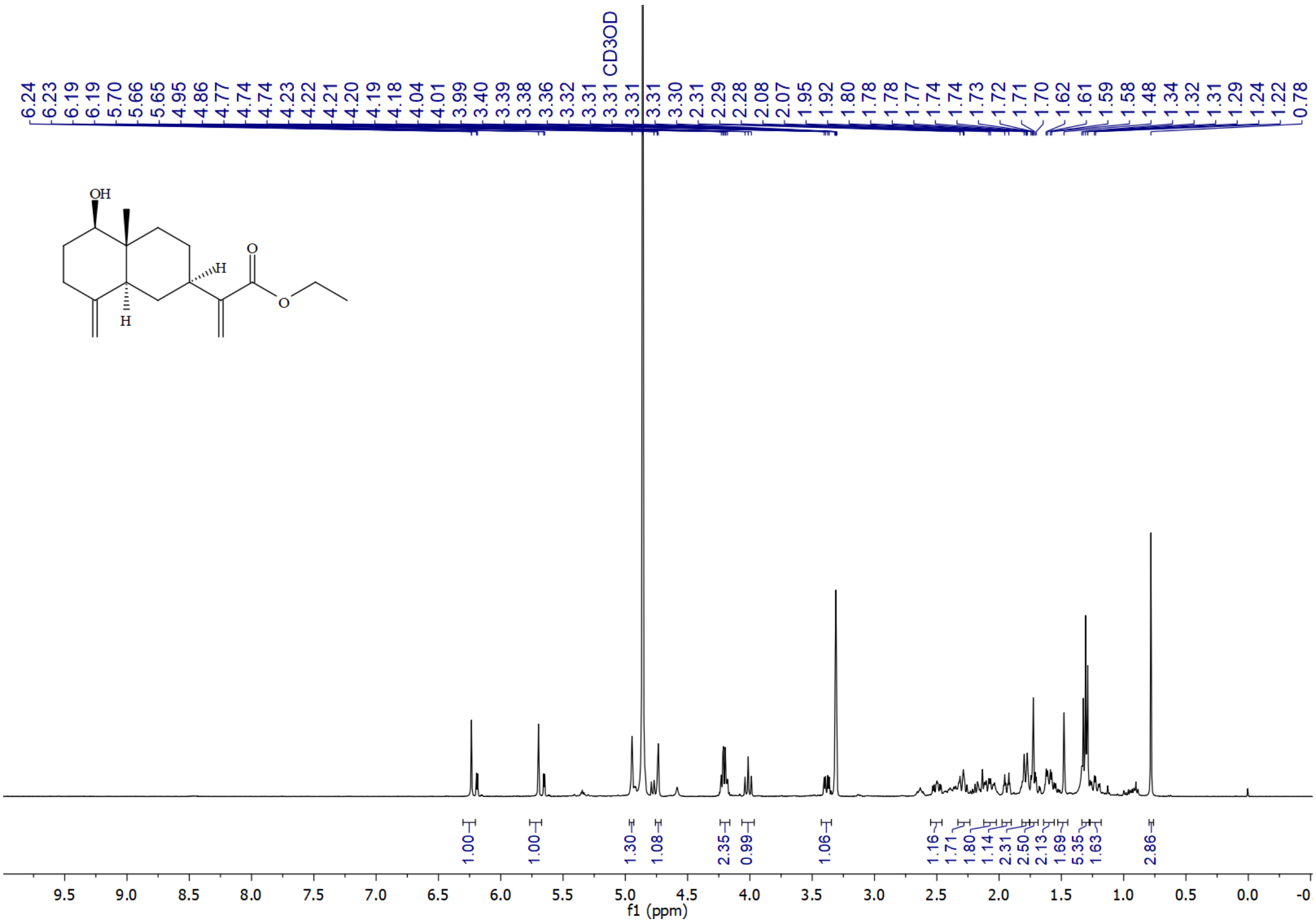

Fig. S32. ^{13}C NMR spectrum of compound **9** in methanol-d_4

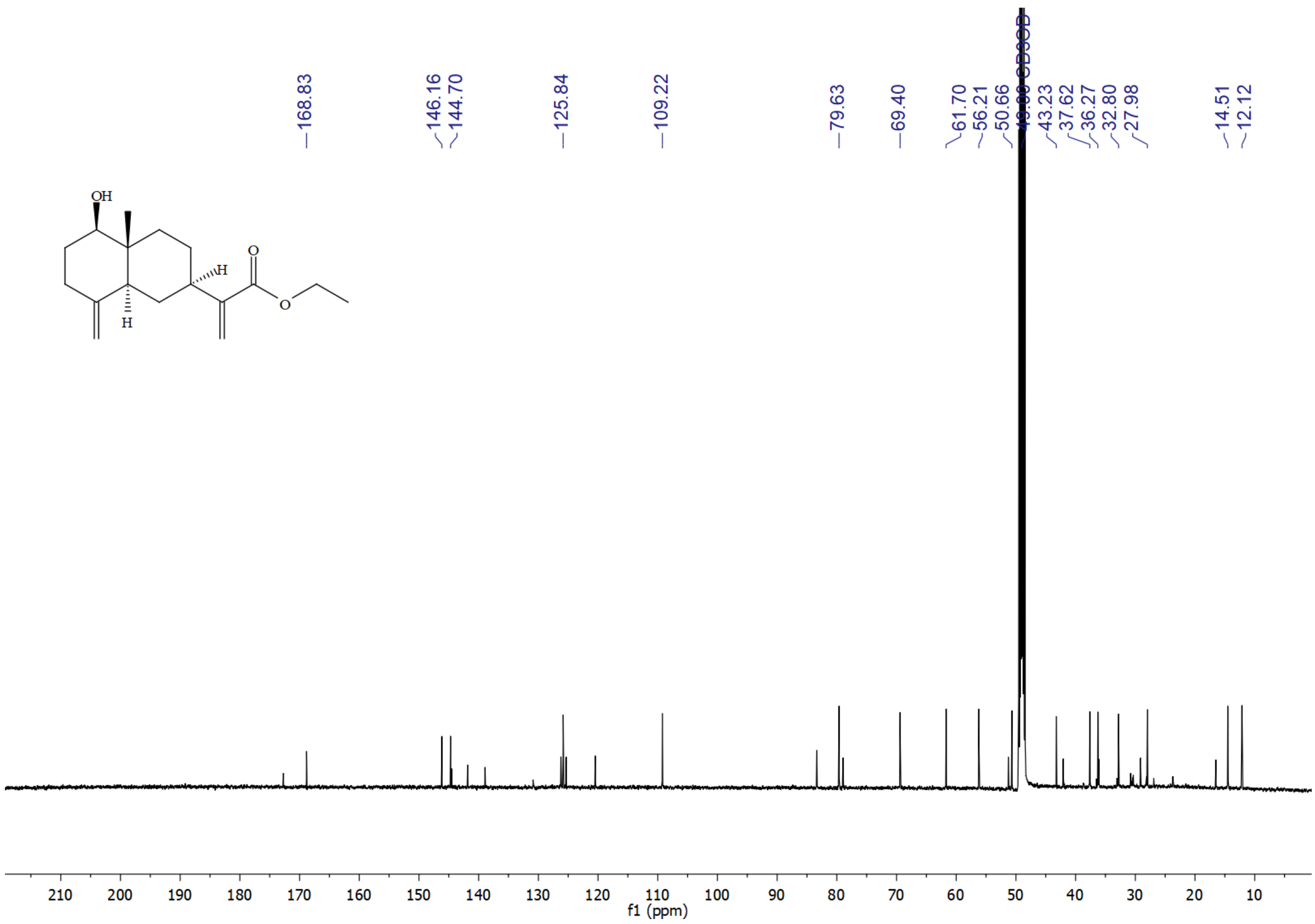

Fig. S33. (±)-ESIMS spectra of compound **9**

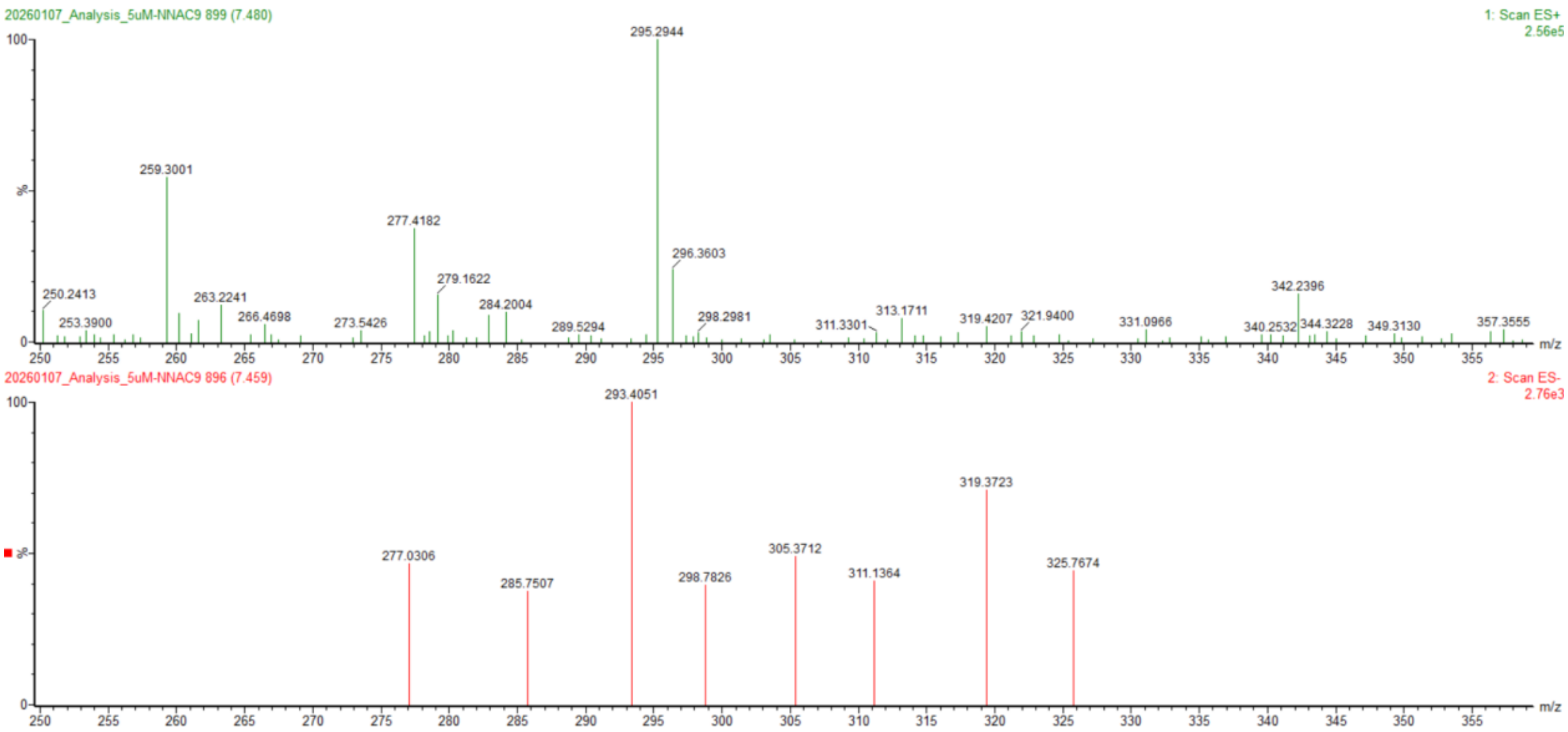

Fig. S34. ^{1}H NMR spectrum of compound **10** in $CDCl_3$

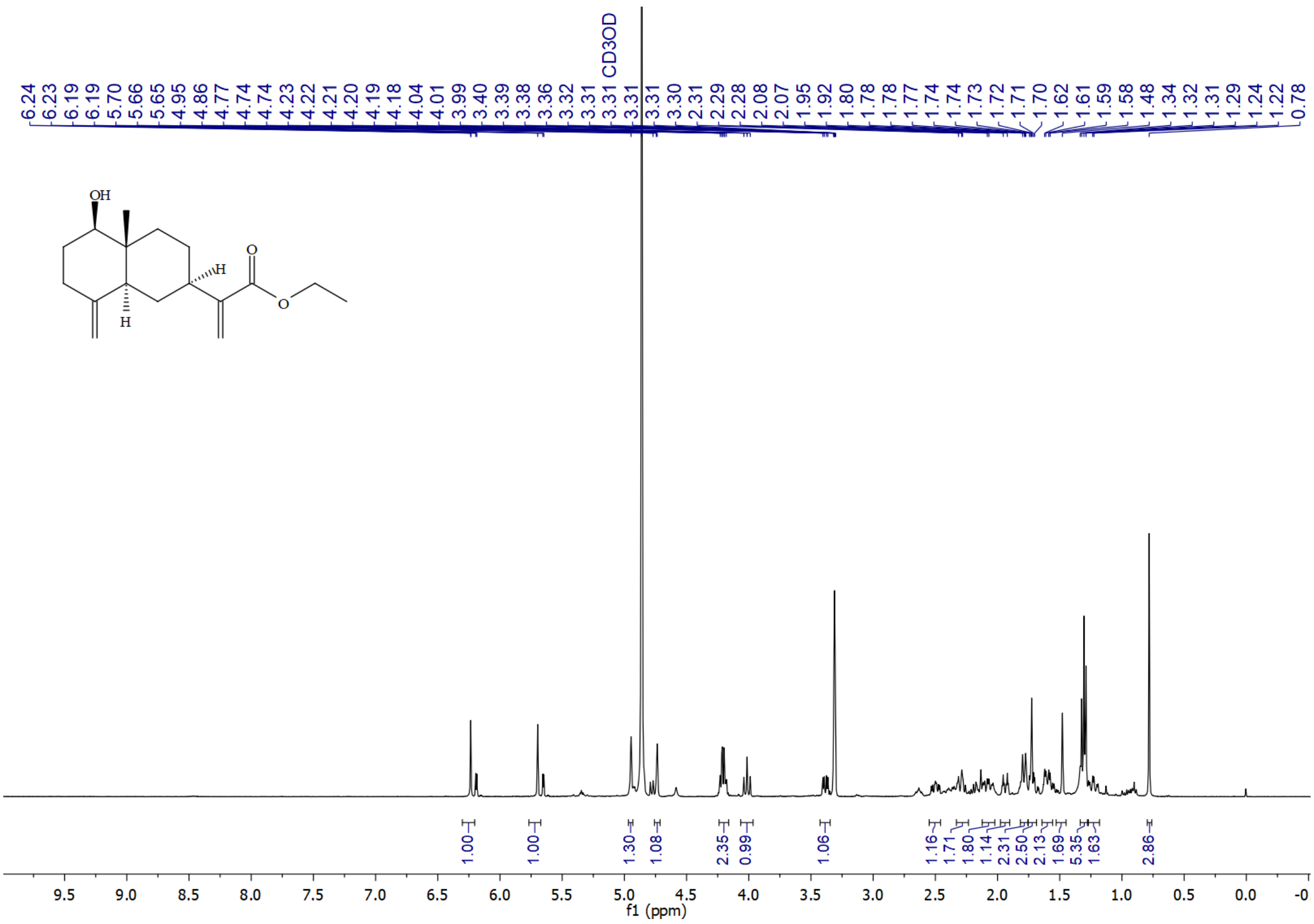

Fig. S35. ^{13}C NMR spectrum of compound **10** in $CDCl_3$

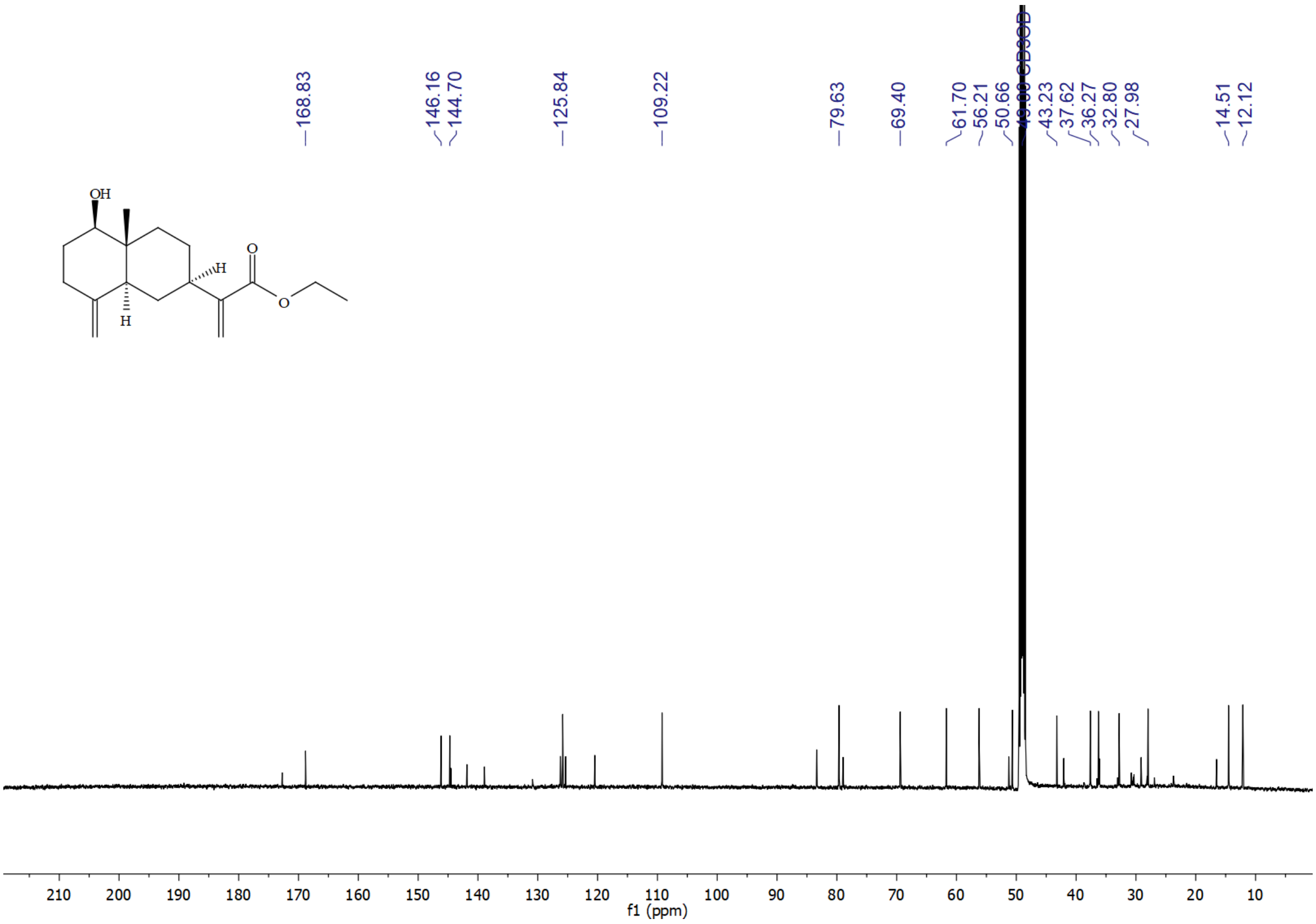

Fig. S36. (±)-ESIMS spectra of compound **10**

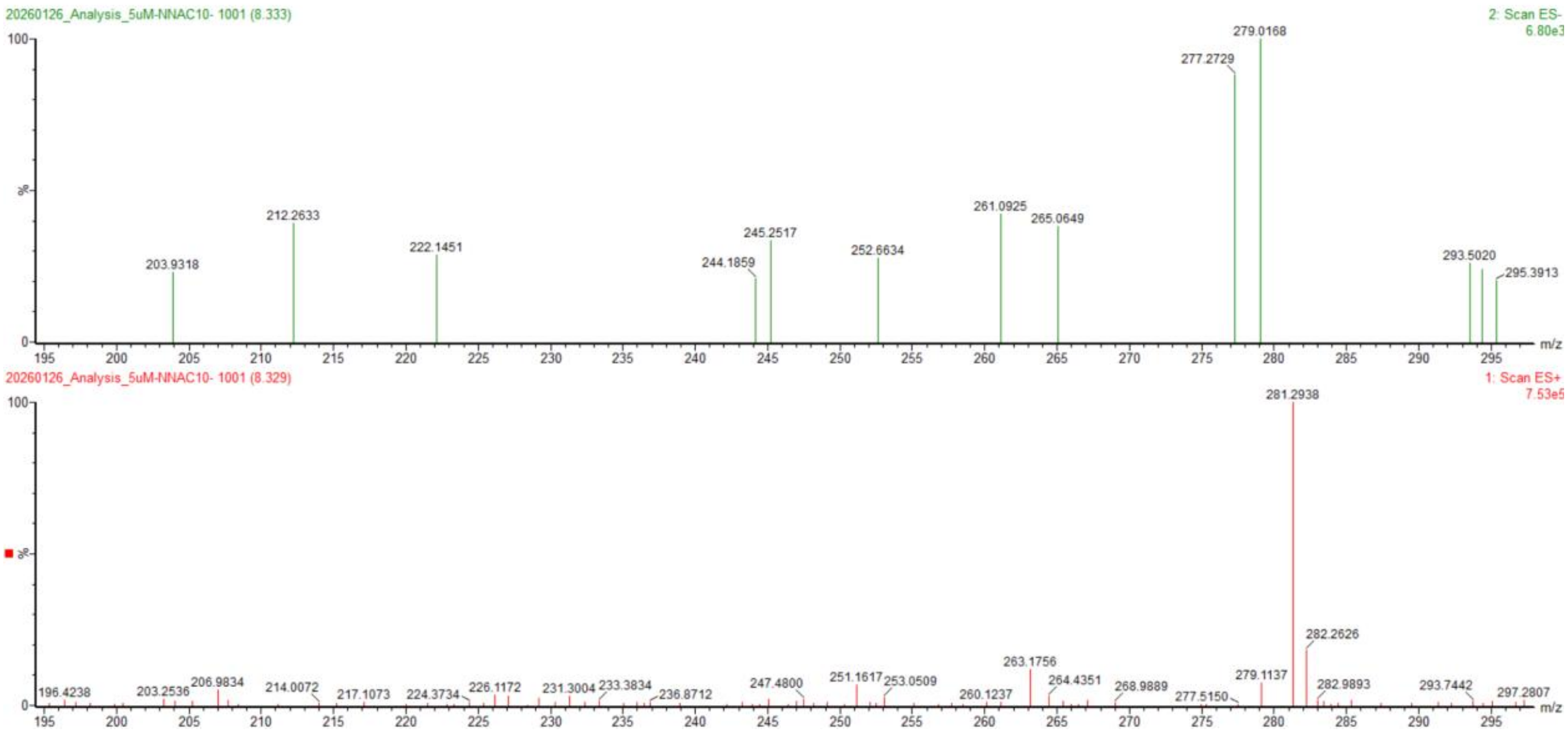

4. Computational Section

Table S3. PCM/ωB97XD/6-311G** Reoptimized Conformers of Compound **1**.

	G (kcal/mol)	ΔG (kcal/mol)	Boltzmann factor	Equilibrium mole fraction
**1-1**	-554927.4737	0	1	0.8818
**1-2**	-554924.2684	3.2053	0.0044	0.0039
**1-3**	-554922.9550	4.5186	0.0004	0.0004
**1-4**	-554926.2620	1.2117	0.1290	0.1138

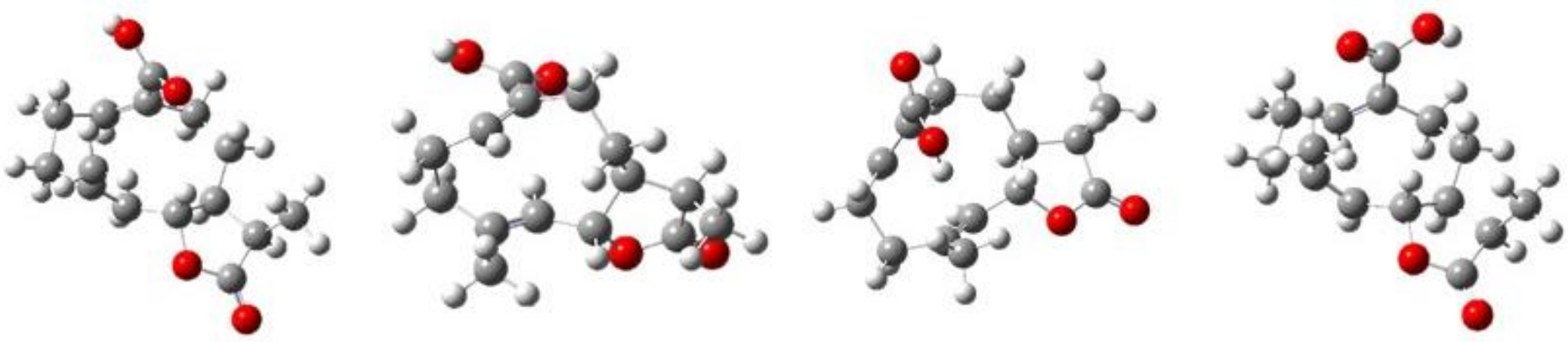

1-1 **1-2** **1-3** **1-4**
